# Population Genomics Reveals Distinct Lineage of the Asian Soybean Rust Fungus *Phakopsora pachyrhizi* in the United States of America Unrelated to Brazilian Populations

**DOI:** 10.1111/mpp.70135

**Published:** 2025-08-07

**Authors:** Everton Geraldo Capote Ferreira, Yoshihiro Inoue, Harun M. Murithi, Tantawat Nardwattanawong, Jitender Cheema, Ruud Grootens, Sirlaine Albino Paes, George Mahuku, Matthieu H. A. J. Joosten, Glen Hartman, Yuichi Yamaoka, M. Catherine Aime, Sérgio H. Brommonschenkel, H. Peter van Esse, Yogesh K. Gupta

**Affiliations:** ^1^ 2Blades Evanston Illinois USA; ^2^ The Sainsbury Laboratory University of East Anglia Norwich UK; ^3^ International Institute of Tropical Agriculture (IITA) Dar es Salaam Tanzania; ^4^ Laboratory of Phytopathology Wageningen University Wageningen the Netherlands; ^5^ Department of Computational and Systems Biology John Innes Centre, Norwich Research Park Norwich UK; ^6^ Departamento de Fitopatologia Universidade Federal de Viçosa Viçosa Brazil; ^7^ Department of Crop Sciences University of Illinois Urbana Illinois USA; ^8^ Institute of Life and Environmental Sciences University of Tsukuba Tsukuba Japan; ^9^ Department of Botany and Plant Pathology Purdue University West Lafayette Indiana USA

**Keywords:** fungicide resistance, lineages, Phakopsoraceae, population structure, Pucciniales, soybean rust

## Abstract

Asian soybean rust (ASR), caused by the obligate biotrophic fungus *Phakopsora pachyrhizi,* was first reported in the continental United States of America (USA) in 2004 and over the years has been of concern to soybean production in the United States. The prevailing hypothesis is that *P. pachyrhizi* spores were introduced into the United States via hurricanes originating from South America, particularly hurricane Ivan. To investigate the genetic diversity and global population structure of *P. pachyrhizi,* we employed exome‐capture based sequencing on 84 field isolates collected from different geographic regions worldwide. We compared the gene‐encoding regions from all these field isolates and found that four major mitochondrial haplotypes are prevalent worldwide. Here, we provide genetic evidence supporting multiple incursions that have led to the currently established *P. pachyrhizi* population of the United States. Phylogenetic analysis of mitochondrial genes further supports this hypothesis. We observed limited genetic diversity in *P. pachyrhizi* populations across different geographic regions, suggesting a clonal population structure. Additionally, this study is the first to report the F129L mutation in the *Cytb* gene outside South America, which is associated with strobilurin tolerance. This study provides the first comprehensive characterisation of *P. pachyrhizi* population structures defined by genetic evidence from populations across major soybean‐growing regions.

## Graphical abstract

Genomic analysis identifies the US population of Asian soybean rust fungus *Phakopsora pachyrhizi* as genetically distinct from South American and East African isolates, offering new insights into its global diversity.

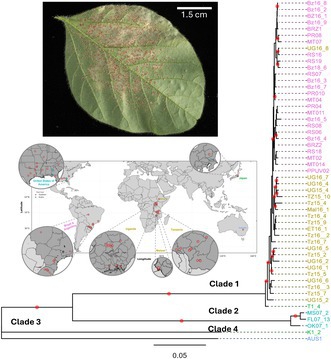

## 1 Introduction

Global food security is threatened by emerging and re‐emerging pests and pathogens (Ristaino et al. 2021). Recent incursions of plant diseases such as wheat blast in Bangladesh (Islam et al. 2016), wheat stem rust in Western Europe (Saunders et al. 2019) and Fusarium wilt caused by Tropical Race 4 in Venezuela and Peru (Acuña et al. 2022) represent serious threats to crop productivity. Currently, 11%–30% of crops are still estimated to be lost because of microbial diseases and pests (Savary et al. 2019). Soybean ( *Glycine max* ) is one of the primary sources of edible oil and plant proteins. Asian soybean rust (ASR), caused by the obligate biotrophic fungus *Phakopsora pachyrhizi*, is a highly destructive disease of soybean (Kelly et al. 2015). In Brazil, the cost of managing ASR was estimated at up to US $2.2 billion during the 2013/2014 growing season (Godoy et al. 2016). However, in recent years, the economic impact of this disease has declined because of the implementation of effective public policies, such as regulated sowing dates, the adoption of a host‐free period, and changes in cropping systems. These measures have helped mitigate environmental conditions favourable for ASR epidemics (Godoy et al. 2016).

*Phakopsora pachyrhizi* was first reported in Japan in 1902 and initially referred to as *Uredo sojae* Henn. (Hennings 1903). It was later formally described as *P. pachyrhizi* Syd. & P. Syd. in 1914 (Sydow and Sydow 1914). Until 1934, the disease was only reported across Asia and Australia (Ono et al. 1992). In 1994, ASR was first reported in Hawaii (Killgore and Heu 1994). From 1996 to 2001, the pathogen was reported in various regions across southern and central Africa (Levy 2005). However, unconfirmed *P. pachyrhizi* occurrences in Africa before 1996 were also reported (Javaid and Ashraf 1978; Haudenshield and Hartman 2015). In 2001, *P. pachyrhizi* was reported in Paraguay and Brazil (Rossi 2003; Yorinori et al. 2005) and the pathogen quickly spread across South America over the following 3 years. However, ASR was not reported in mainland United States until 2004, when it was reported for the first time in Louisiana (Stokstad 2004; Schneider et al. 2005). Shortly after this, the presence of *P. pachyrhizi* was detected across the Southeast United States in Alabama, Arkansas, Florida and Mississippi, and it was perceived as a serious threat to soybean production in those regions (Stokstad 2004). Aerobiological model simulations implicated hurricanes, in particular hurricane Ivan, as the most likely mode of introduction of *P. pachyrhizi* urediniospores, originating from South America, into the continental United States in 2004 (Isard et al. 2005, 2007; Pan et al. 2006). Unfavourable disease conditions, such as the harsh winter climate, along with meticulous monitoring, were critical in limiting the impact of the disease (Sikora 2014; Kelly et al. 2015).

Previous studies reporting the genetic diversity of *P. pachyrhizi* populations worldwide have shown a lack of genetic differentiation and population structure between populations from South America, particularly Brazil and Argentina (Jorge et al. 2015; Darben et al. 2020; da Rocha et al. 2024) and Nigeria (Twizeyimana et al. 2011). On the contrary, this limited genetic diversity does not necessarily correlate with a low diversity of virulence profiles within these populations of *P. pachyrhizi*. In fact, several unique and shared pathotypes have been identified across different countries including Brazil, Argentina and Paraguay (Akamatsu et al. 2013, 2017), the United States (Walker et al. 2011; Paul et al. 2015), Kenya, Malawi, and Nigeria (Twizeyimana et al. 2009; Murithi et al. 2017, 2021), Uruguay (Stewart et al. 2019; Larzábal et al. 2022), Mexico (García‐Rodríguez et al. 2022) and Bangladesh (Hossain and Yamanaka 2019). Interestingly, studies of early *P. pachyrhizi* isolates collected from the 2000's in the United States (Zhang et al. 2012) and Brazil (Freire et al. 2008) revealed a high genetic diversity during the initial outbreaks of ASR. These two studies suggest multiple ribotypes (a pattern of ribosomal RNA bands, to detect polymorphism) in Brazilian populations, originating from Africa and Asia, whereas US populations contained ribotypes from South America, Africa, Asia and Australia. These findings suggest that early *P. pachyrhizi* populations in both Brazil and the United States were established through multiple introductions. Although all previous studies had used molecular markers (SSRs and AFLP markers), as well as housekeeping gene sequences (rDNA internal transcribed spacer [ITS] and *ADP*), these approaches have their own advantages and limitations (Rush et al. 2019; Sheeja et al. 2021). More importantly, although these housekeeping genes are useful for assessing species‐level genetic variation and phylogenetic relationships, they do not provide a broad picture of the genetic differences at the whole genome level. Therefore, inferences on the global migration of *P. pachyrhizi* on the basis of these analyses might be incomplete.

The 1.25 Gb genomes of three *P. pachyrhizi* isolates were recently sequenced (Gupta et al. 2023), facilitated by advances in long‐read sequencing technologies. The *P. pachyrhizi* genome, characterised by its large size, high repeat content (93% transposable elements), significant heterozygosity, and the dikaryotic nature of infectious urediniospores, has historically presented significant challenges for genome assembly and robust population genomic analyses. The availability of these high‐quality genome assemblies now enables population studies of *P. pachyrhizi* with much greater resolution, comparable to what has been applied to other plant pathogens. New approaches such as field‐pathogenomics have emerged as a powerful approach for pathogen diagnostics, surveillance, evolutionary analysis and analysis of population structure of plant pathogens, as has been performed for wheat stripe rust, wheat powdery mildew, wheat blast and Fusarium head blight (Hubbard et al. 2015; Kelly and Ward 2018; Jouet et al. 2019; Thilliez et al. 2019; Radhakrishnan et al. 2019).

In this study, we used an exome capture‐based sequencing approach to understand the global spread and evolutionary dynamics of *P. pachyrhizi*. We studied the genetic diversity and evolutionary relationships among the populations of *P. pachyrhizi*. To this end, we applied phylogenetic and population‐genetic approaches to a geographically diverse set of field isolates collected from East Africa, South America, North America, Asia and Australia. We identified distinct mitochondrial haplotypes (mt‐haplotypes) distributed across different geographic regions worldwide. Unexpectedly, our phylogenetic analyses indicate that the *P. pachyrhizi* population in the United States forms a distinct clade compared to the population in Brazil and East Africa. We demonstrate that the current view on the spread of *P. pachyrhizi* is still incomplete and does not support a sole South American hypothesis for the origin of ASR in the United States, but rather highlights a complex migration dynamic of *P. pachyrhizi* populations.

## 2 Results

**FIGURE 1:**
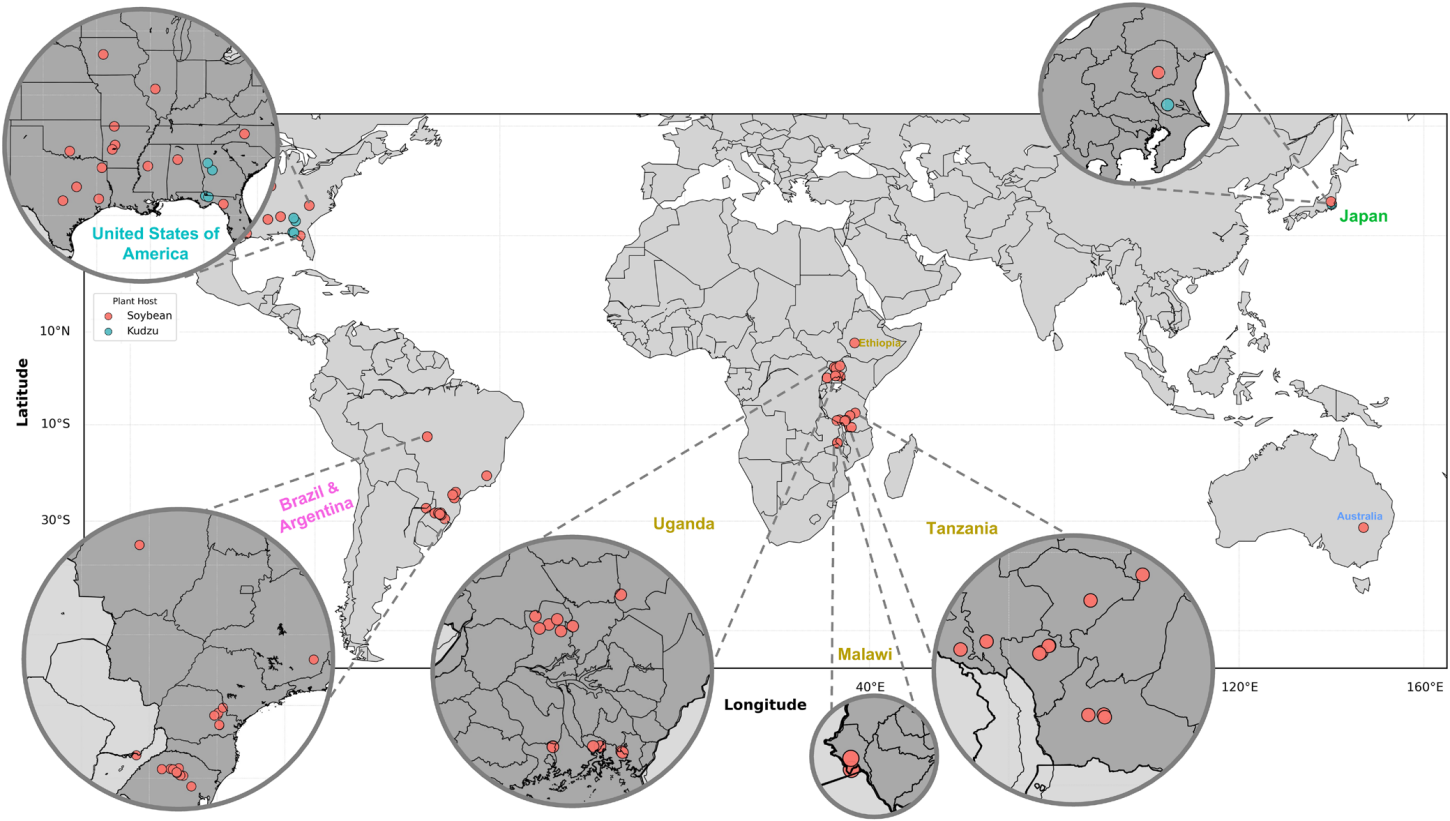
Sampling locations of *Phakopsora pachyrhizi* analysed by exome‐capture sequencing. Dots on the map represent the locations and numbers of the samples collected. The colour corresponds to the host where the samples/isolates originated from.

To estimate the genetic diversity of *P. pachyrhizi*, infected soybean samples were collected from different geographic regions (Figure 1; Table S1). A bait library was designed to capture *P. pachyrhizi* gene sequences from total DNA extracted from the infected samples on the basis of RNA‐seq data from a time course study of the UFV02 isolate of *P. pachyrhizi* (Gupta et al. 2023). In total, 84 field isolates were sequenced, and an uninfected soybean cv. Williams 82 (W82) leaf sample was included to assess the specificity of the baits. All field isolates were collected from infected soybeans, except four field isolates from the United States and the Japanese K1‐2 isolate (Yamaoka et al. 2014), which were collected from kudzu ( *Pueraria lobata* ), a wild legume host of *P. pachyrhizi*.

**FIGURE 2:**
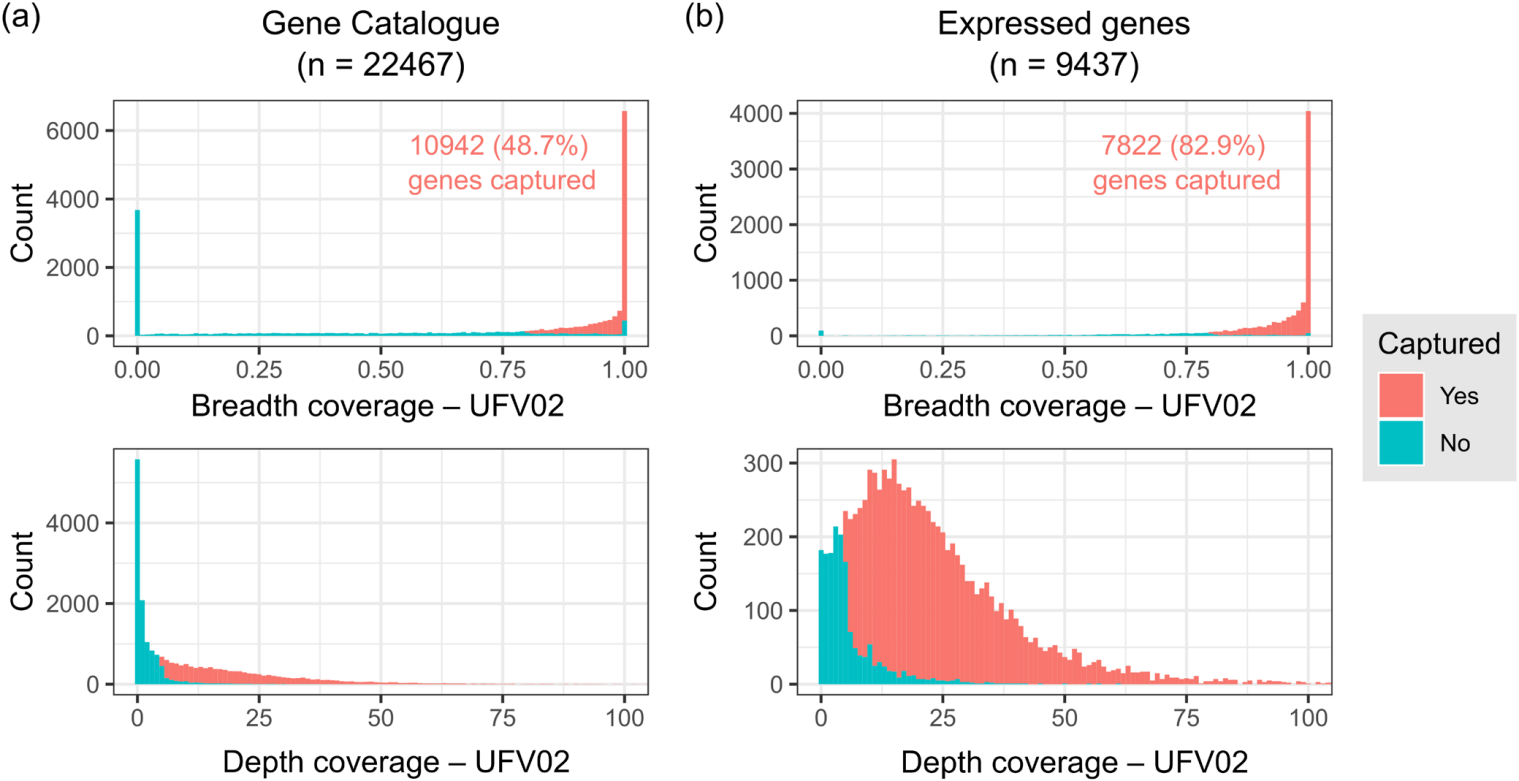
Evaluation of the gene space coverage by exome‐capture sequencing of *Phakopsora pachyrhizi*. Histograms representing the breadth (upper panel) and depth (lower panel) coverage of *P. pachyrhizi* genes by exome‐capture sequencing reads of isolate UFV02. The whole gene set (UFV02 “Gene Catalogue”) and a set of expressed genes were evaluated in (a) and (b), respectively. A gene was judged “captured” if the gene had a breadth coverage of over 0.8 and a depth coverage of over 5.

To evaluate the performance of the bait library, we mapped the reads of UFV02 to its reference genome assembly (Gupta et al. 2023). The UFV02 genome contains 22,467 annotated genes, of which 10,942 were captured (Figure 2a). This outcome was anticipated, as the bait library was specifically designed to target only expressed genes. We captured 7822 out of 9437 expressed genes described in the UFV02 transcriptome, suggesting that 82.9% of the expressed genes from UFV02 were successfully captured using the exome‐capture based method (Figure 2b). We then assessed the sequence read coverage for the 10,942 captured genes across all 84 field samples and from the uninfected leaf of soybean W82. As expected, no *P. pachyrhizi* reads were detected in the W82 control, demonstrating the high specificity of the exome‐capture method for *P. pachyrhizi* DNA sequences. We observed differences in read depth and coverage in the nuclear genome, as well as in the mitochondrial genome across all the samples, which is probably related to the severity level of *P. pachyrhizi* infection (Figures S1 and S2).

### 2.1 Distinct Mt‐Haplotypes Reveal the Population Structure in *P. pachyrhizi* at the Geographic Level

Mitochondrial genomes evolve independently from nuclear genomes, and polymorphisms in introns and intergenic regions of conserved mt genes are often used to assess the genetic diversity within populations (Ballard and Whitlock 2004; Pantou et al. 2008; Kim et al. 2016; Jiménez‐Becerril et al. 2018). To evaluate the polymorphisms in the mt genomes of the field isolates, we mapped their reads to the mt genome of *P. pachyrhizi*. We aligned the mt genomes (31,825 bp) of the Taiwan‐72‐1 isolate (Stone et al. 2010) and the MT2006 isolate (Gupta et al. 2023) (Figure S3). Despite being sequenced using different sequencing technologies—Sanger sequencing for Taiwan‐72‐1 and PacBio for MT2006—the alignment revealed a high level of genetic conservation between these two isolates. To validate the polymorphic sites, we mapped the sequencing reads from a subset of field isolates to both reference genomes and compared the number of detected variants, along with the counts of mapped and unmapped reads. In both cases, we consistently identified the same single‐nucleotide polymorphisms (SNPs) across samples, with identical mapping statistics. Given the availability of the Taiwan‐72‐1 genome and its annotation in GenBank, we selected this genome as the reference for all subsequent analyses.

**FIGURE 3:**
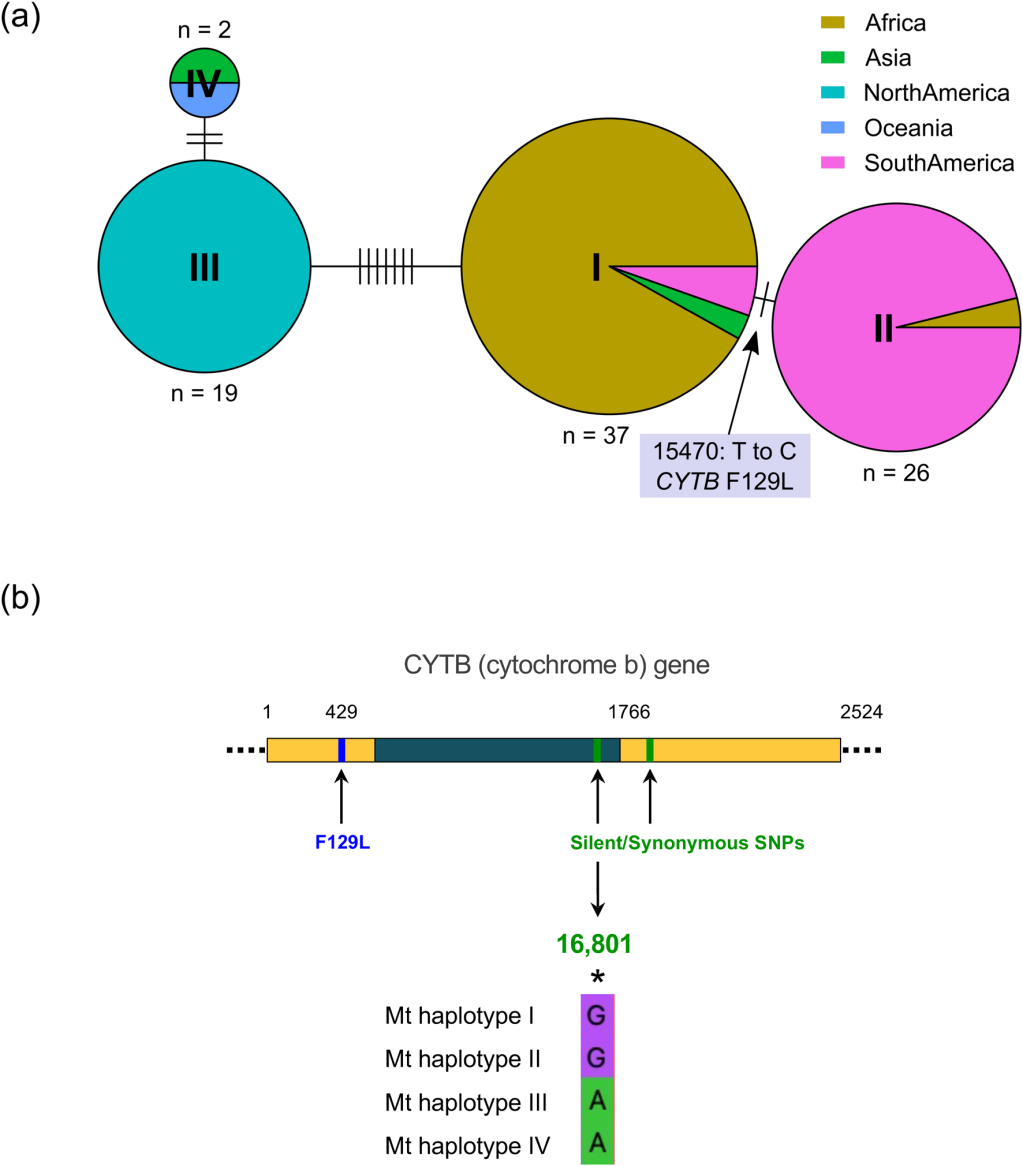
Mt‐haplotype network inferred from nucleotide polymorphisms in the mt genome of *Phakopsora pachyrhizi*. (a) Pie charts represent mt‐haplotypes and their geographic origins. The size of each node is proportional to the number of isolates sharing that mt‐haplotype. Connecting edges indicate genetic relationships, with each tick mark representing a single single‐nulceotide polymorphim (SNP) or InDel. (b) A non‐synonymous SNP (F129L) associated with resistance to quinone outside inhibitor (QoI) fungicides is marked by a blue vertical bar. Synonymous SNPs (no amino acid change) are marked by green vertical bars. Genotypes at SNP marker 16,801 (converted to KASP assay) in four mt‐haplotypes are shown below.

Although the *P. pachyrhizi* mt genome is highly conserved across all field isolates, we identified six SNPs and four InDels between the isolates. On the basis of those variants, four major mt‐haplotypes were observed across the 84 sequenced field isolates (Figure 3a). Notably, a non‐synonymous SNP marker (a T to C change at position 15,470) resulting in an F129L mutation in the cytochrome b (*Cytb*) gene was identified. This mutation, predominantly found in field isolates from South America, is exclusive to mt‐haplotype II (Figure 3a). This F129L mutation, previously reported in South America, has been linked to reduced sensitivity to quinone outside inhibitor fungicides (QoIs) (Klosowski, May De Mio, et al. 2016; Müller et al. 2021). Remarkably, this mutation was also observed in two field isolates from Uganda, rendering this the first report of the F129L mutation in *P. pachyrhizi* outside South America (Figure 3a).

Surprisingly, all field isolates from the United States clustered into a single mt‐haplotype, mt‐haplotype III, exhibiting no intragenic variation. The US field isolates showed seven polymorphic sites (alternative alleles) compared to those from South America and East Africa (Figure 3a). We identified two unique mutations (synonymous SNPs) in the *Cytb* gene, shared among all the US field isolates, the Australian isolate AUS1, and the K1‐2 isolate from Japan, distinguishing mt‐haplotype III and IV from mt‐haplotypes I (mainly African field isolates) and II (mostly South America) (Figure 3b). This suggests that the current US population emerged from a single, distinct lineage separate from the South American and East African *P. pachyrhizi* populations. The low genetic variation observed in the mt genome indicates limited genetic exchange between populations, as expected, considering that *P. pachyrhizi* spreads through clonally produced urediniospores. Lastly, the mt‐haplotype structure reveals geographic separation of *P. pachyrhizi* populations, with only exceptions of two Japanese isolates harbouring distinct mt‐haplotypes.

### 2.2 Unique Origin of the *P. pachyrhizi* Lineage in the United States

**FIGURE 4:**
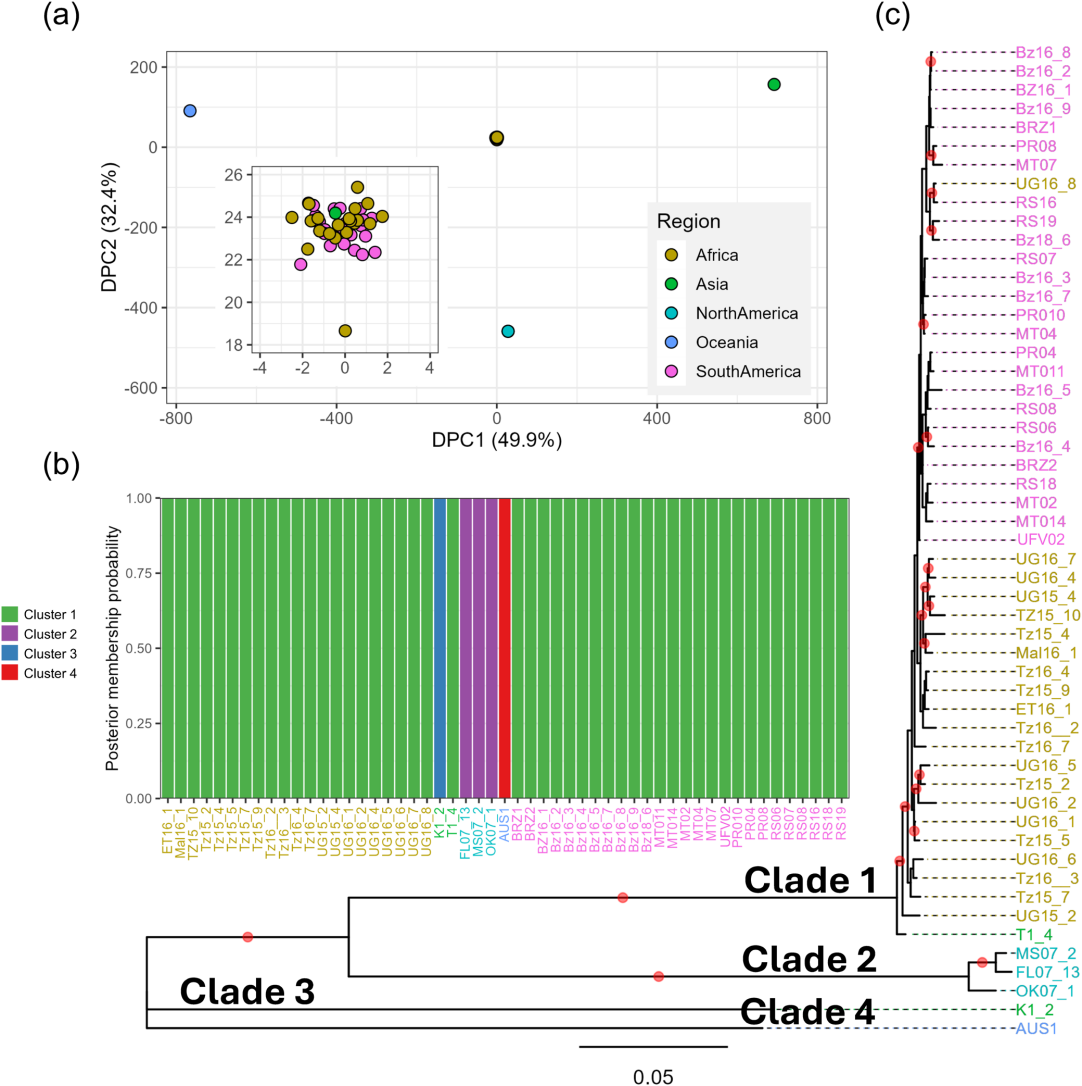
Population structure and phylogenetic relationships of *Phakopsora pachyrhizi* samples inferred from nuclear genome single‐nucleotide polymorphisms (SNPs). (a) Discriminant analysis of principal components (DAPC) using SNP markers from 53 samples, with principal components 1 and 2 plotted. (b) Probability of population membership is shown on the *y*‐axis for each sample, grouped in bins along the *x*‐axis. The stacked bar chart represents assigned population membership probabilities, with clusters distinguished by different colours. (c) Phylogenetic tree inferred from 33,634 SNP markers. Nodes with bootstrap support greater than 80% are marked with red dots.

To elucidate the population structure of *P. pachyrhizi*, we mapped the sequencing reads from all the field isolates to the UFV02 reference genome assembly. On the basis of read depth coverage, we selected 53 field isolates and identified 33,634 high‐confidence SNP markers to define the population structure and identify genetically related *P. pachyrhizi* isolates. Several US samples exhibited low sequencing depth because of lesser but sufficient sample quality. Three high‐quality US samples were included in the population structure analysis of *P. pachyrhizi* (Figure S1a). These selected samples had a read‐depth coverage comparable to field isolates from Brazil and East Africa (Figure S1a). We conducted a discriminant analysis of principal components (DAPC), which revealed four distinct *P. pachyrhizi* populations. An unsupervised genotype clustering analysis further corroborated the presence of these four well‐supported groups (Figures 4a,b and S5).

To assess evolutionary relationships among field isolates and to determine the extent of clonality, we constructed a maximum‐likelihood (ML) phylogenetic tree using the 33,634 biallelic SNP markers, which cover over 7418 expressed genes. The phylogenetic analysis grouped the *P. pachyrhizi* field isolates into two major clades and separate branches for K1‐2 and AUS1 isolates (Figure 4c), consistent with the clusters identified in the DAPC (Figure 4a). A complementary neighbour‐net phylogenetic network further supported the differentiation of *P. pachyrhizi* populations into four distinct groups (Figure S6). The largest clade, Clade 1, includes all the field isolates from South America and East Africa, as well as one of the Japanese isolates, T1‐4. Within Clade 1, two major subgroups, mostly corresponding to their geographic origin, were observed, suggesting a limited genetic exchange between *P. pachyrhizi* populations in South America and East Africa, and implying that these populations may be evolving independently.

All US field isolates formed a distinct clade, Clade 2, separate from Clade 1, with strong bootstrap support (> 80%) (Figure 4c). Given the high degree of heterozygosity observed in the *P. pachyrhizi* genome, we assessed heterozygosity across all field isolates (Figure S4). Notably, US field isolates, the Australian isolate AUS1, and the Japanese kudzu isolate K1‐2, exhibited higher heterozygosity (17.2%–28.0% of the SNP sites showed a heterozygous genotype) compared to other field isolates (6.4%–12.6% of the SNP sites showed heterozygous genotype; Figure S4b).

To investigate potential recombination within the populations, we performed linkage disequilibrium (LD) decay analysis on all 53 isolates, as well as on the South American and East African subpopulations. Because of the limited number of isolates from the United States (*n* = 3), we combined them with those from Australia and Japan to enable robust statistical analysis. LD decay, measured as the physical distance at which *r* ^*2*^ declines to 50% of its maximum value (Table S2; Figure S7), was used as an indicator of the frequency of sexual reproduction across populations (Nieuwenhuis and James 2016). In both the full dataset of 53 isolates and the South America subpopulation, LD decay dropped to half its maximum value at ~100 kb. In contrast, the East African subpopulation and the combined United States/Japan/Australia group showed slower decay rates, reaching 50% at around 190 and 228 kb, respectively. These patterns are consistent with those observed in other predominantly asexual fungal species, suggesting limited recombination within the studied populations.

These findings, combined with mt‐haplotype analysis, suggest that the *P. pachyrhizi* population in the United States is not genetically linked to the South American population and suggest no recent movement of *P. pachyrhizi* between these regions. Additionally, our phylogenetic analysis revealed that the K1‐2 isolate (collected from the kudzu host in Japan) and the AUS1 isolate (collected from soybean in Australia) diverged significantly from all the other sequenced isolates, forming separate clades (clade 3 and clade 4, respectively). These results suggest that *P. pachyrhizi* populations in Japan and Australia are highly divergent and warrant further investigations with larger sample sizes from the region.

For the exome capture study, we used US field isolates collected in 2007–2008, and two samples from Japan (T1‐4 and K1‐2) collected in 2007. To investigate the genetic relationship with more recent *P. pachyrhizi* populations in the United States (2017, *n* = 19), Japan (2012–2017, *n* = 22) and Puerto Rico (2015, *n* = 2), we converted two SNPs on the mitochondrial *Cytb* gene that efficiently distinguished the mt‐haplotypes I and II, and mt‐haplotypes III and IV (Figure 3) into kompetitive allele‐specific PCR (KASP) assays (Table S3). One of the KASP markers (COB2_2, 16,801) showed clear allelic discrimination and was kept for further analyses. All the recent US field isolates carried the same allele as the earlier US field isolates from 2007, indicating a consistent mt‐haplotype III not linked to the South American population (Table S4). Similarly, most Japanese field isolates (except one) shared the same allele as present in the US populations and the K1‐2 isolate. Interestingly, both Puerto Rican field isolates carried the alternate allele, consistent with the South American mt‐haplotype II. In summary, the KASP assay COB2_2 developed here provides a robust tool for genotyping *P. pachyrhizi* mt‐haplotypes. It offers a valuable resource for tracking pathogen movement and understanding the global population structure of this important soybean pathogen.

## 3 Discussion

Current population studies of *P. pachyrhizi* have primarily relied on simple‐sequence repeat (SSR) markers, amplified fragment length polymorphisms (AFLPs), and single‐locus sequencing of genes, such as ITS and ADP‐ribosylation factor (ARF) (Anderson et al. 2008; Zhang et al. 2012; Twizeyimana et al. 2011). Although these methods have provided valuable insights, they lack the resolution needed to capture fine‐scale population structure and genetic diversity. Our analyses suggest low levels of genetic diversity within Brazilian and East African populations, whereas *P. pachyrhizi* populations in the United States appear genetically divergent from both the Brazilian and East African populations. Although these neutral markers provide valuable baseline data on population differentiation, they offer limited insights into the adaptive genetic structure needed for understanding evolutionary dynamics and pathogenic variability at the genome level. In contrast, whole‐genome or reduced representation sequencing approaches offer a higher resolution strategy for studying adaptive evolution and population diversity, especially in understudied pathogens (Hubbard et al. 2015; Jouet et al. 2019; Thilliez et al. 2019).

Like other rust species, *P. pachyrhizi* is an obligate biotrophic pathogen, dependent on living host tissue for growth and propagation. This inability to grow in vitro presents significant challenges and limitations for genetic studies and other investigations (Panstruga 2003; Lorrain et al. 2019). Additionally, the large genome size of *P. pachyrhizi* (1.25 Gb) and its high repeat content (93% of TEs) further complicate large‐scale population studies (Gupta et al. 2023). These challenges stem from the substantial resources required to isolate high‐molecular‐weight DNA, the high costs of sequencing, and the complexity of analysing such repetitive and heterogeneous genomic data. To overcome these limitations, we developed an exome‐capture‐based approach that selectively targets genic regions. This approach has been extensively used for genetic studies in plants (King et al. 2015; Mukrimin et al. 2018; Hussain et al. 2018; Dong et al. 2020), human pathogens (Carpi et al. 2015; Quek and Ng 2024), animals (McClure et al. 2014; Fairfield et al. 2015) and plant pathogens such as *Phytophthora infestans* (Thilliez et al. 2019; Coomber et al. 2024). Using the recently sequenced *P. pachyrhizi* genome as a scaffold, we validated the specificity and efficacy of this approach, successfully capturing 83% of all expressed genes in a proof‐of‐concept study. This significantly enhanced our ability to draw accurate inferences on pathogen migration and population structure of *P. pachyrhizi*.

### 3.1 Distinct Mt‐Haplotypes and Global Lineages of *P. pachyrhizi*

Through mt genome analysis, we identified four distinct mt‐haplotypes among *P. pachyrhizi* field isolates. A single non‐synonymous mutation, F129L, in the *Cytb* gene distinguished mt‐haplotypes I and II, with mt‐haplotype II carrying the F129L mutation in 95% of the South American field isolates. The reduced efficiency of various fungicide classes in controlling ASR in Brazil has been associated with the increasing prevalence of known mutations in fungicide target genes (Godoy 2012; Godoy et al. 2022). In particular, the F129L mutation is associated with reduced sensitivity to QoI fungicides (Godoy et al. 2016) and has been previously observed at high frequencies in South American *P. pachyrhizi* populations (Klosowski, Brahm, et al. 2016; Klosowski, May De Mio, et al. 2016; Müller et al. 2021; Claus et al. 2022). Notably, we detected the F129L mutation in two field isolates from Uganda, making it the first report of this mutation outside South America. Although strobilurin‐based fungicides remain effective against ASR in African countries such as Uganda and Ethiopia (Kawuki et al. 2003; Abebe et al. 2022), the detection of this mutation underscores the need for continuous monitoring.

Interestingly, US field isolates formed a unique mt‐haplotype, mt‐haplotype III, with seven unique polymorphic sites compared to mt‐haplotypes I and II. The *P. pachyrhizi* field isolates from the United States analysed in this study did not exhibit the F129L mutation. This absence is likely due to the relatively low ASR incidence in the United States, which reduces the need for fungicide applications and, consequently, the selective pressure for this mutation (Bradley et al. 2021). This genetic uniqueness suggests limited selection pressure for QoI resistance, as ASR outbreaks in the United States are sporadic and often constrained by climatic factors. This represents the first report identifying a unique genetic signature in US *P. pachyrhizi* populations, suggesting a distinct evolutionary trajectory.

The emergence of new races and pathotypes in plant‐pathogenic fungi often depends on their mutation and recombination rates (Wyka et al. 2022; Amezrou et al. 2024). In *P. pachyrhizi*, long‐distance dispersal via airstreams can promote genetic exchange, particularly when spores land on an alternate host. This process can significantly influence population substructure, even in geographically separated regions. Our phylogenetic reconstruction revealed two major clades: Clade 1, comprising isolates from South America and Africa, and Clade 2, exclusively containing US isolates. The clustering of South American and African field isolates into Clade 1 supports the previous hypothesis that Brazilian *P. pachyrhizi* populations likely originated from Africa (Freire et al. 2008). Our results showed that, despite its airborne nature, the genetic similarity between populations from these two regions suggests a predominantly clonal structure, likely facilitated by similar tropical climates and asexual reproduction (Goellner et al. 2010).

In contrast, the US population, represented by Clade 2, exhibited significant genetic divergence, potentially driven by the overwintering of *P. pachyrhizi* on alternative plant hosts such as kudzu in regions like Florida and the Gulf Coast, where milder conditions prevail (https://soybean.ipmpipe.org/soybeanrust/). During harsh winters in the US Midwest, the absence of soybean as a host is mitigated by the presence of kudzu, which provides refuge for this obligate pathogen. However, the harsh winter conditions likely impose a bottleneck effect, reducing the survival of *P. pachyrhizi* spores and further shaping its population structure (Harmon et al. 2005; Isard et al. 2005, 2007; Kelly et al. 2015). Each year, airborne spores are carried by wind from southern regions to the Midwest, but their late arrival in the growing season reduces the likelihood of severe ASR outbreaks. This annual spore movement, combined with the pathogen's dependence on kudzu as a winter reservoir, likely contributes to the genetic differentiation of US *P. pachyrhizi* populations from those in South America and Africa.

Additionally, multiple independent introductions of *P. pachyrhizi* into the continental United States may have further shaped the genetic structure of these populations. It is plausible that *P. pachyrhizi* populations of Asian origin entered the United States, potentially via Hawaii and subsequently adapted to infect soybean and kudzu, while surviving the harsh winter conditions. Notably, Japan experiences severe winters, which may explain the shared genetic adaptations observed between Japanese and US isolates (Twizeyimana and Hartman 2012; Yamaoka et al. 2014). Our results further support this hypothesis as one of the Japanese isolates, K1‐2, and the US isolates from kudzu shared the same mutation in the *Cytb* mitochondrial gene.

A recent study by da Rocha et al. (2024) also identified two major clades within *P. pachyrhizi* populations, corresponding to older and more recent lineages. However, their study was limited by the inclusion of only two US isolates: one from Hawaii (HW94‐1, isolated in 1994) and one from the continental United States (FL07‐01, isolated in 2007). Interestingly, FL07‐01 clustered closely with isolates from Africa and Brazil, whereas HW94‐1 clustered with older isolates from India, Taiwan and Australia (da Rocha et al. 2024). The limited sampling may have underestimated the genetic diversity and population structure within the United States. However, it is plausible that during the initial years of *P. pachyrhizi*'s colonisation in the continental United States, multiple introductions occurred, with FL07‐01 potentially representing a South American lineage that later diminished as populations introduced via Hawaii became dominant.

Our findings challenge the long‐held assumption that US ASR outbreaks stemmed exclusively from hurricane‐driven spore dispersal from Brazil. Instead, the genetic distinctiveness and clustering of the US *P. pachyrhizi* population within Clade 2 suggest that *P. pachyrhizi* introduction into the United States may have occurred through independent migration events, rather than a single introduction from Brazil, as previously proposed by Zhang et al. (2012).

In addition, isolates from Australia (AUS1) and Japan (K1‐2) formed highly divergent clades compared to clades 1 and 2, suggesting substantial genetic differentiation in these populations. The high genetic diversity observed in the US population is unlikely to arise from sexual reproduction, as no alternate host has been identified to date. It is most likely that kudzu, a widely distributed alternative host, along with the harsh winters, has driven adaptation in the US population, enabling it to effectively infect both soybean and kudzu. We observed higher heterozygosity in isolates from the United States, K1‐2 and AUS1, when compared to those from Brazil and East Africa. In rust fungi such as *Puccinia striiformis* f. sp. *tritici*, variation in heterozygosity has been linked to recent recombination or cryptic sexual reproduction (Schwessinger et al. 2020; Zheng et al. 2013). Although a sexual stage has not been confirmed in *P. pachyrhizi*, our findings raise the possibility of rare recombination events or somatic hybridisation. Alternatively, heterozygosity may result from long‐term clonal propagation in a dikaryotic organism, with divergence between nuclei over time. These scenarios could contribute to the pathogen's adaptability and warrant further investigation.

Interestingly, despite the clonal nature of the *P. pachyrhizi* populations highlighted by the low diversity across different countries, a high diversity in virulence profiles has been consistently reported (Twizeyimana et al. 2011; Twizeyimana and Hartman 2012; Yamaoka et al. 2014; Zhang et al. 2012). Therefore, this indicates genetic forces acting in the selection and generation of different races of the pathogen. Additionally, the role of transposable elements in fungal genome evolution cannot be overlooked. Transposable elements are known to contribute to adaptive variations in traits such as pathogenicity and virulence (Torres et al. 2021; Fouché et al. 2022; Oggenfuss and Croll 2023). With approximately 93% of the *P. pachyrhizi* genome consisting of transposable elements, of which ~12% is being expressed (Gupta et al. 2023), it is plausible that these play a critical role in generating genetic variability in the absence of sexual reproduction and somatic hybridisation. Recent studies have pointed out the role of tramsposable elements in shaping genetic variability in various fungal species. For instance, transposable elements mediate metal resistance in *Paecilomyces variotii* (Urquhart et al. 2022); drive strain‐specific evolution and host‐specific expression regulation of transposable elements in *Rhizophagus irregularis* (Oliveira and Corradi 2024); and contribute to lineage‐specific differences in *Magnaporthe oryzae* infecting various grasses (Nakamoto et al. 2023). Although somatic hybridisation has been documented in other rust fungi, it is a mechanism that generates genetic diversity through the exchange of nuclei between different lineages (Li et al. 2019; Sperschneider et al. 2023), but has not yet been confirmed in *P. pachyrhizi*. However, a study reported hyphal anastomosis between urediniospore germ tubes in *P. pachyrhizi*, facilitating nuclear migration into a shared hyphal network (Vittal et al. 2012). This observation raises the possibility of nuclear exchange and recombination. Although somatic hybridisation does not generate new mutations, it can create novel allele combinations by bringing together two genetically distinct nuclei, potentially driving the rapid emergence of new *P. pachyrhizi* races.

### 3.2 Implications for Disease Management

Our exome capture‐based sequencing approach provided an in‐depth view of genetic variation within the genic regions of *P. pachyrhizi*, enabling the identification of SNP markers associated with the distinct geographic origins. Such genetic markers can readily be converted into low‐cost KASP assays for use in‐field monitoring and early detection of fungicide‐resistant or more virulent *P. pachyrhizi* populations. Such methodologies will be crucial for enabling on‐time targeted interventions, as is being carried out in the case of other important plant pathogens (Bueno‐Sancho et al. 2017; Radhakrishnan et al. 2019; Paineau et al. 2024). These methods have great potential for future applications in the detection of resistance‐breaking isolates and in monitoring the durability of disease resistance in the field.

Overall, this study contributes to the understanding of *P. pachyrhizi* population dynamics worldwide and highlights the emergence of a genetically divergent lineage among isolates from the United States, which is likely driven through multiple independent introductions into the country. Exome‐capture sequencing technology offers a valuable tool for studying the adaptive genetic variation of this pathogen. Future research should delve into the role of transposable elements in generating diversity in the absence of sexual reproduction or alternate host. Additionally, the knowledge of host–plant interactions, such as kudzu and soybean, under varying climate conditions, will be necessary for a more comprehensive understanding of the evolutionary dynamics of *P. pachyrhizi*. These will be important in devising management strategies that can help reduce the continued threat of this highly adaptive and destructive pathogen.

## 4 Experimental Procedures

### 4.1 Field Isolates Collection

Eighty‐one ASR‐infected field isolates from different geographic regions, including Australia (*n* = 1), Argentina (1), Brazil (25), East Africa (35), and the United States (19) (Table S1) were collected. The field isolates from East Africa were collected from Ethiopia, Malawi, Uganda and Tanzania between 2015 and 2016. The isolates from Brazil were collected in 2015 and 2016, and the US isolates were collected in 2007 and 2008. The Brazilian isolate UFV02 (collected in 2006) and the Japanese isolates T1‐4 (from cultivated soybean) and K1‐2 (from kudzu, *P. lobata* ), collected in 2007, were also included in the analysis (Gupta et al. 2023; Yamaoka et al. 2014). Spore suspension and inoculation assay were carried out as previously described to obtain infected leaf material for the three isolates. Briefly, spores of the isolates were heat‐shocked at 40°C for 5 min, suspended in an aqueous solution (0.01% Tween 20), and mixed thoroughly, and the concentration was adjusted to approximately 5 × 10^4^ spores/mL with a haemocytometer. Four‐week‐old soybean plants (cv. Williams 82) were sprayed on the abaxial surface of the leaves with the spore suspension, kept at 100% relative humidity in the dark for 24 h. After 24 h, inoculated plants were kept at 22°C and 70% relative humidity, with a 16 h photoperiod. 14 days after inoculation (dai), infected leaves were collected and stored at −80°C until DNA extraction. The infection was performed in three biological replicates, and for each replicate, three leaves per plant (trifoliates) and three plants were used for each individual replicate.

### 4.2 Exome Capture Design, Library Preparation and Sequencing

Genomic DNA from all 84 infected leaf samples (100 mg of leaf tissue) was extracted using the DNeasy Plant Mini Kit (Qiagen) according to the manufacturer's instructions. The DNA samples were provided to Arbor Biosciences (Ann Arbor, Michigan, US) for library preparation, target capture enrichment and Illumina sequencing. Briefly, the target capture baits library was built on the basis of the *P. pachyrhizi* UFV02 transcriptome dataset publicly available (Gupta et al. 2023). The probe (also referred here as baits) sequence candidates were designed following the resistance gene enrichment sequencing (RenSeq) pipeline previously established (Jupe et al. 2013). The probe sequences were designed over and between exon‐exon boundaries of the retrieved expressed gene sequences. The first probe started at the leftmost nucleotide and followed the predicted coding direction of the gene. The target probe sequences were 100 bp in length, 4× tiling, and 25 bp overlap between baits. In total, around 162,000 baits were designed on the basis of %GC, secondary propensities, cross‐complementarity, and 80% identity between baits, and further synthesised by Arbor Biosciences (Ann Arbor, MI, USA). Genomic DNA from the 84 samples was subjected to target capture hybridization using the biotinylated bait library. Enriched libraries were submitted for sequencing on an Illumina HiSeq 4000 platform, 150 bp paired‐end reads. As a positive control, cv. Williams 82 infected with UFV02 was considered, and as a negative control, DNA of non‐inoculated cv. Williams 82 was subjected to hybridization and sequencing to confirm the cross‐hybridisation of baits.

### 4.3 Filtering Steps and Variant Calling

The quality of the raw reads from sequencing was checked using the FastQC program (https://www.bioinformatics.babraham.ac.uk/projects/fastqc/). Low quality base reads were trimmed using Trimmomatic v. 0.39 with default options (Bolger et al. 2014). The paired‐end reads were mapped to the *P. pachyrhizi* (UFV02 v2.1) reference genome downloaded from the Joint Genome Institute (JGI) (https://mycocosm.jgi.doe.gov/PpacPPUFV02/PpacPPUFV02.info.html) using BWA v. 0.7.17 (Li and Durbin 2009) with the BWA‐MEM algorithm and default parameters. Resulting SAM files were sorted and converted into BAM files using SAMtools v. 1.9 (Danecek et al. 2021). The filtered paired‐end reads were also mapped to the *P. pachyrhizi* mitochondrial genome (Taiwan 72–1 isolate, GenBank: GQ332420), converted to the BAM format and sorted as mentioned above. BAM files generated using nuclear and mitochondrial genomes were used independently to generate variant files for follow‐up phylogenetic and population analyses.

For variant calling, PCR duplicates were marked in the BAM files using the Picard v. 1.118 toolkit (http://broadinstitute.github.io/picard/) and variant calling was performed using the GATK in two rounds (van der Auwera and O'Connor 2020). First, variants (SNPs and InDels) from all the samples were predicted by the HaplotypeCaller function; the resulting VCF was split into two files using the SelectVariants function, one for SNPs and another for InDels. Then, the VariantFiltration function was applied for hard filtering SNPs with quality thresholds (QD < 2.0; FS > 60.0; MQ < 40.0; MQRankSum < −12.5; ReadPosRankSum < −8.0) and for filtering InDels (QD < 2.0; FS > 200.0; ReadPosRankSum < −20.0; SOR > 10.0). Base quality scores in the BAM files were recalibrated using the filtered variants files with the BaseRecalibrator and ApplyBQSR functions. Finally, the recalibrated BAM files were used for the second variant calling using the HaplotypeCaller and GenotypeGVCFs functions.

### 4.4 Phylogenetics and Population Structure Analyses

Only biallelic SNPs from the nuclear genome (missing data < 10%) were used for the phylogenetic analyses. Maximum‐likelihood trees were inferred using IQ‐TREE (v. 2.2.2.7, Minh et al. 2020); the best‐scoring tree from 10 independent runs was selected, and branch supports were obtained by ultrafast bootstrap (Hoang et al. 2018) with 1000 replicates. The tree was visualised by the ggtree package (v. 3.10.0, Yu et al. 2016) on RStudio (2023.09.1 + 494) with R (v. 4.3.2). The population structure was analysed using DAPC with the Adegenet package (v. 2.1.10) on an R environment, and the optimal number of genetic clusters was estimated by using Bayesian information criterion (BIC) values. Variants from the mitochondrial genome were used to build a mt‐haplotype network using the HaploNet function in the *pegas* package (v. 1.3) in an R environment. The mt‐haplotypes were coloured on the basis of the geographic origin and visualised by the *ggplot2* package (v. 3.4.4) in R.

Breadth and depth of coverage were calculated for all the genes captured by the library. Briefly, breadth of coverage corresponds to the percentage of target bases with at least one mapped read (1× coverage). The proportion of heterozygous/homozygous SNPs for each sample was calculated by bcftools v. 1.9 (Danecek et al. 2021).

Linkage disequilibrium (LD) decay was calculated as the decline of the squared correlation (*r* ^*2*^) between pairs of SNP markers with increasing physical distance, using PopLDdecay (v. 3.43) (Zhang et al. 2019). The same filtered file previously used for population structure analyses was used for the LD analysis. Only biallelic SNPs with a minor allele frequency (MAF) of ≥ 1% were included. This dataset was used for *r* ^*2*^ calculation, applying a 300‐kb maximum physical distance between two SNP pairs. Pairwise *r* ^*2*^ values were extracted for all SNP pairs calculated by PopLDdecay. LD values were smoothed using non‐overlapping bins (1 kb by default) to reduce noise in sparse datasets. We then focused on estimating two commonly used LD decay metrics: LD_50_^1^ and LD_50_^2^ (Nieuwenhuis and James 2016). LD_50_^1^ is defined as the physical distance (in base pairs) at which LD (*r* ^2^) declines to 50% of its maximum value. LD_50_^2^ is the distance at which *r* ^*2*^ reaches the midpoint between the maximum and minimum LD values observed in the dataset.

### 4.5 Mt‐Haplotype Analysis and KASP Validation

In order to develop mt SNP markers able to distinguish between ASR subpopulations, we selected two SNP sites for marker development. Primers for KASP markers were designed using an in‐house Python script and used for genotyping of an extra set of ASR field isolates collected in 2017 from Louisiana, United States. DNA was extracted from approximately 100 mg of *P. pachyrhizi s*oybean infected leaves with the DNeasy Plant Mini kit (Qiagen) following the manufacturer's protocol. DNA samples were diluted to a concentration of 10 ng/μL and submitted to KASP reactions using PACE allele‐specific master mix (3CR Bioscience), following the manufacturer's guidelines. Briefly, 1.8 μL of diluted DNA was dried into a pellet, after which 2.4 μL of KASP reaction mix was added (1.2 μL ultrapure water, 1.2 μL PACE mix, 0.03 μL primers mix). The PCR programme used is a touchdown PCR, and the thermocycling steps conditions were the following: 94°C for 15 min followed by 10 cycles of 94°C for 20 s and then 65°C–75°C for 1 min, an additional 45 cycles of 94°C for 20 s and 57°C for 1 min. Fluorescent endpoint reading was performed on a Tecan Safire plate reader, and the data analysis was performed by Klustercaller software (v. 2.22.0.5, LGC).

## Author Contributions

E.G.C.F., Y.I., H.M.M., T.N., J.C., R.G., G.H., Y.Y., M.C.A., S.H.B., H.P.v.E. and Y.K.G. performed research. E.G.C.F., Y.I., H.M.M., T.N., J.C., R.G., S.A.P. and Y.K.G. analysed the data. E.G.C.F., H.P.v.E. and Y.K.G. edited the manuscript. E.G.C.F. and Y.K.G. wrote the paper. G.M., M.H.A.J.J., S.H.B., H.P.v.E. and Y.K.G. directed aspects of the project.

## Conflicts of Interest

The authors declare no conflicts of interest.

## Supporting information


**Figure S1:** Read breadth and depth coverage of the UFV02 “Gene Catalogue” genes in 85 exome‐capture samples. (a) Read breadth and (b) depth coverage over genic regions of 10,942 protein‐coding genes in the nuclear genome (exome‐captured UFV02 “Gene Catalogue” genes) were calculated and are represented as boxplots. The first sample is W82 (red), a negative control to test the specificity of the exome‐capture array. Outlier points were omitted. Dots indicate mean values.

**Figure S2:** Read breadth coverage of mt genes in 85 exome‐capture samples. (a) Read breadth coverage over genic regions of 15 protein‐coding genes in the mt genome (b) Log_10_ fold depth coverage of sequencing reads. The first sample is W82 (red), a negative control to test the specificity of the exome‐capture array. Both sequencing read breadth and depth coverage were calculated and are represented as boxplots.

**Figure S3:** Genomic comparison between mitochondrial genomes of the isolates Taiwan‐72 and MT2006. (a) Whole genome alignment performed by Geneious software (MAUVE alignment) showing highly conservation between the Taiwan‐72 genome (Genbank CG332420) and the MT2006 genome. (b) Number of mapped reads across different mt reference genomes in samples from South America (Bz_16_9; Bz_18_6; MT014; MT07; PR04, UFV02); Africa (Tz15_5; Tz16_7; UG15_2; UG16_7); United States of America (Fl07_13; MS07_2; OK07_1); Australia (AUS1); and Japan (K1‐2; T1‐4). Screenshots of IGV software showing that the mutation F129L present in Brazilian isolates is identified using both the (c) Taiwan‐72 and (d) MT2006 reference genomes.

**Figure S4:** Read breadth coverage and genotypes of SNP sites in the nuclear genome in 53 exome‐capture samples.

**Figure S5:** Elbow plot of Bayesian Information Criterion (BIC) for different number of clusters, *k*. To identify the optimal number of clusters, k‐means was run sequentially with increasing values of *k*, and different clustering solutions were compared using BIC.

**Figure S6:** A neighbour‐net phylogenetic network of *P. pachyrhizi* samples inferred from nuclear genome SNPs. A phylogenetic network was inferred from 33,634 SNP markers with hamming distance and neighborNet function implemented in the phangorn package in R. Dots represent the samples and are colour‐coded on the basis of the geographic origin of the samples.

**Figure S7:** Linkage disequilibrium (LD) patterns in the full set of 53 isolates of *P*. *pachyrhizi*. Squared correlation coefficient (*r* ^2^) values are represented in the *y*‐axis and physical distance between pairs of SNPs are represented in the *x*‐axis. (a) LD decay patterns of the full set of 53 isolates. (b) South America sub‐set calculated with 26 isolates. (c) LD decay patterns of Africa sub‐set (21 isolates). (d) LD decay of United States, Japan and Australia isolates (5 isolates).

**Table S1:** Information regarding the samples used for the exome‐capture.

**Table S2:** Linkage disequilibrium metrics calculated for the full dataset and sub‐populations.

**Table S3:** Synonymous mutations converted to the SNP markers sequences and KASP assay sequences.

**Table S4:** Genotyping results from the KASP assay COB2_2 in the new set of samples from USA and Japan.

## Data Availability

The raw sequencing data have been deposited at NCBI under the accession number PRJEB83226.

## References

[mpp70135-bib-0001] Abebe, A. T. , K. Belachew , M. Hailemariam , Y. Sileshi , and A. Ortega‐Beltran . 2022. “Interaction of Varieties and Fungicides Across Seasons and Locations for the Control of Asian Soybean Rust (*Phakopsora pachyrhizi*) in Southwestern Ethiopia.” Crop Protection 158: 106008.

[mpp70135-bib-0002] Acuña, R. , M. Rouard , A. M. Leiva , et al. 2022. “First Report of *Fusarium oxysporum* f. sp. *cubense* Tropical Race 4 Causing Fusarium Wilt in Cavendish Bananas in Peru.” Plant Disease 106: PDIS09211951PDN.

[mpp70135-bib-0003] Akamatsu, H. , N. Yamanaka , R. M. Soares , et al. 2017. “Pathogenic Variation of South American *Phakopsora pachyrhizi* Populations Isolated From Soybeans From 2010 to 2015.” Japan Agricultural Research Quarterly: JARQ 51: 221–232.

[mpp70135-bib-0004] Akamatsu, H. , N. Yamanaka , Y. Yamaoka , et al. 2013. “Pathogenic Diversity of Soybean Rust in Argentina, Brazil, and Paraguay.” Journal of General Plant Pathology 79: 28–40.

[mpp70135-bib-0005] Amezrou, R. , A. Ducasse , J. Compain , et al. 2024. “Quantitative Pathogenicity and Host Adaptation in a Fungal Plant Pathogen Revealed by Whole‐Genome Sequencing.” Nature Communications 15: 1933. 10.1038/s41467-024-46191-1 PMC10908820 38431601

[mpp70135-bib-0006] Anderson, S. J. , C. L. Stone , M. L. Posada‐Buitrago , et al. 2008. “Development of Simple Sequence Repeat Markers for the Soybean Rust Fungus, *Phakopsora pachyrhizi* .” Molecular Ecology Resources 8: 1310–1312. 10.1111/j.1755-0998.2008.02272.x. 21586030

[mpp70135-bib-0007] Ballard, J. W. O. , and M. C. Whitlock . 2004. “The Incomplete Natural History of Mitochondria.” Molecular Ecology 13: 729–744. 15012752 10.1046/j.1365-294x.2003.02063.x

[mpp70135-bib-0008] Bolger, A. M. , M. Lohse , and B. Usadel . 2014. “Trimmomatic: A Flexible Trimmer for Illumina Sequence Data.” Bioinformatics 30: 2114–2120. 24695404 10.1093/bioinformatics/btu170 PMC4103590

[mpp70135-bib-0009] Bradley, C. A. , T. W. Allen , A. J. Sisson , et al. 2021. “Soybean Yield Loss Estimates due to Diseases in the United States and Ontario, Canada, From 2015 to 2019.” Plant Health Progress 22: 483–495.

[mpp70135-bib-0010] Bueno‐Sancho, V. , D. C. E. Bunting , L. J. Yanes , K. Yoshida , and D. G. O. Saunders . 2017. “Field Pathogenomics: An Advanced Tool for Wheat Rust Surveillance.” In Wheat Rust Diseases: Methods and Protocols, edited by S. Periyannan , 13–28. Springer. 10.1007/978-1-4939-7249-4_2 28856637

[mpp70135-bib-0011] Carpi, G. , K. S. Walter , S. J. Bent , A. G. Hoen , M. Diuk‐Wasser , and A. Caccone . 2015. “Whole Genome Capture of Vector‐Borne Pathogens From Mixed DNA Samples: A Case Study of *Borrelia burgdorferi* .” BMC Genomics 16: 434. 26048573 10.1186/s12864-015-1634-x PMC4458057

[mpp70135-bib-0012] Claus, A. , K. Simões , and L. L. M. De Mio . 2022. “SdhC‐I86F Mutation in *Phakopsora pachyrhizi* Is Stable and Can Be Related to Fitness Penalties.” Phytopathology 112: 1413–1421. 35080435 10.1094/PHYTO-10-21-0419-R

[mpp70135-bib-0013] Coomber, A. , A. Saville , and J. B. Ristaino . 2024. “Evolution of *Phytophthora infestans* on Its Potato Host Since the Irish Potato Famine.” Nature Communications 15: 6488. 10.1038/s41467-024-50749-4 PMC11300821 39103347

[mpp70135-bib-0014] da Rocha, V. D. , E. G. C. Ferreira , F. M. Castanho , et al. 2024. “The Genetic Diversity of the Soybean Rust Pathogen *Phakopsora pachyrhizi* Has Been Driven by Two Major Evolutionary Lineages.” 10.1101/2024.10.03.616435. 40319936

[mpp70135-bib-0016] Danecek, P. , J. K. Bonfield , J. Liddle , et al. 2021. “Twelve Years of SAMtools and BCFtools.” GigaScience 10: giab008. 33590861 10.1093/gigascience/giab008 PMC7931819

[mpp70135-bib-0017] Darben, L. M. , A. Yokoyama , F. M. Castanho , et al. 2020. “Characterization of Genetic Diversity and Pathogenicity of *Phakopsora pachyrhizi* Mono‐Uredinial Isolates Collected in Brazil.” European Journal of Plant Pathology 156: 355–372.

[mpp70135-bib-0019] Dong, C. , L. Zhang , Z. Chen , et al. 2020. “Combining a New Exome Capture Panel With an Effective varBScore Algorithm Accelerates BSA‐Based Gene Cloning in Wheat.” Frontiers in Plant Science 11: 1249. 32903549 10.3389/fpls.2020.01249 PMC7438552

[mpp70135-bib-0020] Fairfield, H. , A. Srivastava , G. Ananda , et al. 2015. “Exome Sequencing Reveals Pathogenic Mutations in 91 Strains of Mice With Mendelian Disorders.” Genome Research 25: 948–957. 25917818 10.1101/gr.186882.114 PMC4484392

[mpp70135-bib-0021] Fouché, S. , U. Oggenfuss , E. Chanclud , and D. Croll . 2022. “A Devil's Bargain With Transposable Elements in Plant Pathogens.” Trends in Genetics 38: 222–230. 34489138 10.1016/j.tig.2021.08.005

[mpp70135-bib-0022] Freire, M. C. M. , L. O. de Oliveira , Á. M. R. de Almeida , et al. 2008. “Evolutionary History of *Phakopsora pachyrhizi* (The Asian Soybean Rust) in Brazil Based on Nucleotide Sequences of the Internal Transcribed Spacer Region of the Nuclear Ribosomal DNA.” Genetics and Molecular Biology 31: 920–931.

[mpp70135-bib-0023] García‐Rodríguez, J. C. , Z. Vicente‐Hernández , M. Grajales‐Solís , and N. Yamanaka . 2022. “Virulence Diversity of *Phakopsora pachyrhizi* in Mexico.” PhytoFrontiers 2: 52–59.

[mpp70135-bib-0024] Godoy, C. 2012. Risk and Management of Fungicide Resistance in the Asian Soybean Rust Fungus Phakopsora pachyrhizi, 87–95. CABI.

[mpp70135-bib-0025] Godoy, C. V. , C. D. S. Seixas , R. M. Soares , F. C. Marcelino‐Guimarães , M. C. Meyer , and L. M. Costamilan . 2016. “Asian Soybean Rust in Brazil: Past, Present, and Future.” Pesquisa Agropecuária Brasileira 51: 407–421.

[mpp70135-bib-0026] Godoy, C. V. , C. Utiamada , M. Meyer , H. Campos , I. Lopes , and A. Tomen . 2022. “Eficiência de Fungicidas Para o Controle da Ferrugem‐Asiática da Soja, *Phakopsora pachyrhizi*, Na Safra 2021/2022: Resultados Sumarizados dos Ensaios Cooperativos.” Embrapa Soja. Circular Tecnica 187: 1–28.

[mpp70135-bib-0027] Goellner, K. , M. Loehrer , C. Langenbach , U. Conrath , E. Koch , and U. Schaffrath . 2010. “ *Phakopsora pachyrhizi*, the Causal Agent of Asian Soybean Rust.” Molecular Plant Pathology 11: 169–177. 20447267 10.1111/j.1364-3703.2009.00589.x PMC6640291

[mpp70135-bib-0028] Gupta, Y. K. , F. C. Marcelino‐Guimarães , C. Lorrain , et al. 2023. “Major Proliferation of Transposable Elements Shaped the Genome of the Soybean Rust Pathogen *Phakopsora pachyrhizi* .” Nature Communications 14: 1835. 10.1038/s41467-023-37551-4 PMC10067951 37005409

[mpp70135-bib-0029] Harmon, P. F. , M. T. Momol , J. J. Marois , H. Dankers , and C. L. Harmon . 2005. “Asian Soybean Rust Caused by *Phakopsora pachyrhizi* on Soybean and Kudzu in Florida.” Plant Health Progress 6: 9.

[mpp70135-bib-0030] Haudenshield, J. S. , and G. L. Hartman . 2015. “Archaeophytopathology of *Phakopsora pachyrhizi*, the Soybean Rust Pathogen.” Plant Disease 99: 575–579. 30699680 10.1094/PDIS-07-14-0772-SR

[mpp70135-bib-0031] Hennings, P. 1903. “Einige Neue Japanische Uredinales.” Hedwigia IV, no. Suppl: 107–108.

[mpp70135-bib-0032] Hoang, D. T. , O. Chernomor , A. von Haeseler , B. Q. Minh , and L. S. Vinh . 2018. “UFBoot2: Improving the Ultrafast Bootstrap Approximation.” Molecular Biology and Evolution 35: 518–522. 10.1093/molbev/msx281. 29077904 PMC5850222

[mpp70135-bib-0033] Hossain, M. M. , and N. Yamanaka . 2019. “Pathogenic Variation of Asian Soybean Rust Pathogen in Bangladesh.” Journal of General Plant Pathology 85: 90–100.

[mpp70135-bib-0034] Hubbard, A. , C. M. Lewis , K. Yoshida , et al. 2015. “Field Pathogenomics Reveals the Emergence of a Diverse Wheat Yellow Rust Population.” Genome Biology 16: 23. 25723868 10.1186/s13059-015-0590-8 PMC4342793

[mpp70135-bib-0035] Hussain, M. , M. A. Iqbal , B. J. Till , and M. Rahman . 2018. “Identification of Induced Mutations in Hexaploid Wheat Genome Using Exome Capture Assay.” PLoS One 13: e0201918. 30102729 10.1371/journal.pone.0201918 PMC6089429

[mpp70135-bib-0036] Isard, S. A. , S. H. Gage , P. Comtois , and J. M. Russo . 2005. “Principles of the Atmospheric Pathway for Invasive Species Applied to Soybean Rust.” Bioscience 55: 851–861.

[mpp70135-bib-0037] Isard, S. A. , J. M. Russo , and A. Ariatti . 2007. “The Integrated Aerobiology Modelling System Applied to the Spread of Soybean Rust Into the Ohio River Valley During September 2006.” Aerobiologia 23: 271–282.

[mpp70135-bib-0038] Islam, M. T. , D. Croll , P. Gladieux , et al. 2016. “Emergence of Wheat Blast in Bangladesh Was Caused by a South American Lineage of *Magnaporthe oryzae* .” BMC Biology 14: 84. 27716181 10.1186/s12915-016-0309-7 PMC5047043

[mpp70135-bib-0039] Javaid, I. , and M. Ashraf . 1978. “Some Observations on Soybean Diseases in Zambia and Occurrence of *Pyrenochaeta glycines* on Certain Varieties.” Plant Disease Reporter 62, no. 1: 46–47.

[mpp70135-bib-0040] Jiménez‐Becerril, M. F. , S. Hernández‐Delgado , M. Solís‐Oba , and J. M. González Prieto . 2018. “Analysis of Mitochondrial Genetic Diversity of *Ustilago maydis* in Mexico.” Mitochondrial DNA Part A, DNA Mapping, Sequencing, and Analysis 29: 1–8. 27728988 10.1080/24701394.2016.1229776

[mpp70135-bib-0041] Jorge, V. R. , M. R. Silva , E. A. Guillin , et al. 2015. “The Origin and Genetic Diversity of the Causal Agent of Asian Soybean Rust, *Phakopsora pachyrhizi*, in South America.” Plant Pathology 64: 729–737.

[mpp70135-bib-0042] Jouet, A. , D. G. O. Saunders , M. McMullan , et al. 2019. “ *Albugo candida* Race Diversity, Ploidy and Host‐Associated Microbes Revealed Using DNA Sequence Capture on Diseased Plants in the Field.” New Phytologist 221: 1529–1543. 30288750 10.1111/nph.15417

[mpp70135-bib-0043] Jupe, F. , K. Witek , W. Verweij , et al. 2013. “Resistance Gene Enrichment Sequencing (RenSeq) Enables Reannotation of the NB‐LRR Gene Family From Sequenced Plant Genomes and Rapid Mapping of Resistance Loci in Segregating Populations.” Plant Journal 76: 530–544. 10.1111/tpj.12307 PMC3935411 23937694

[mpp70135-bib-0044] Kawuki, R. S. , E. Adipala , and P. Tukamuhabwa . 2003. “Yield Loss Associated With Soya Bean Rust (*Phakopsora pachyrhizi* Syd.) in Uganda.” Journal of Phytopathology 151: 7–12.

[mpp70135-bib-0045] Kelly, A. C. , and T. J. Ward . 2018. “Population Genomics of *Fusarium graminearum* Reveals Signatures of Divergent Evolution Within a Major Cereal Pathogen.” PLoS One 13: e0194616. 29584736 10.1371/journal.pone.0194616 PMC5870968

[mpp70135-bib-0046] Kelly, H. Y. , N. S. Dufault , D. R. Walker , et al. 2015. “From Select Agent to an Established Pathogen: The Response to *Phakopsora pachyrhizi* (Soybean Rust) in North America.” Phytopathology 105: 905–916. 25775102 10.1094/PHYTO-02-15-0054-FI

[mpp70135-bib-0047] Killgore, E. , and R. Heu . 1994. “First Report of Soybean Rust in Hawaii.” Plant Disease 78: 1216.

[mpp70135-bib-0048] Kim, J.‐O. , K.‐Y. Choi , J.‐H. Han , I.‐Y. Choi , Y.‐H. Lee , and K. S. Kim . 2016. “The Complete Mitochondrial Genome Sequence of the Ascomycete Plant Pathogen *Colletotrichum acutatum* .” Mitochondrial DNA Part A, DNA Mapping, Sequencing, and Analysis 27: 4547–4548. 26539901 10.3109/19401736.2015.1101556

[mpp70135-bib-0049] King, R. , N. Bird , R. Ramirez‐Gonzalez , et al. 2015. “Mutation Scanning in Wheat by Exon Capture and Next‐Generation Sequencing.” PLoS One 10: e0137549. 26335335 10.1371/journal.pone.0137549 PMC4559439

[mpp70135-bib-0050] Klosowski, A. C. , L. Brahm , G. Stammler , and L. L. M. De Mio . 2016. “Competitive Fitness of *Phakopsora pachyrhizi* Isolates With Mutations in the CYP51 and CYTB Genes.” Phytopathology 106: 1278–1284. 27359265 10.1094/PHYTO-01-16-0008-R

[mpp70135-bib-0051] Klosowski, A. C. , L. L. May De Mio , S. Miessner , R. Rodrigues , and G. Stammler . 2016. “Detection of the F129L Mutation in the Cytochrome b Gene in *Phakopsora pachyrhizi* .” Pest Management Science 72: 1211–1215. 26296393 10.1002/ps.4099

[mpp70135-bib-0052] Larzábal, J. , M. Rodríguez , N. Yamanaka , and S. Stewart . 2022. “Pathogenic Variability of Asian Soybean Rust Fungus Within Fields in Uruguay.” Tropical Plant Pathology 47: 574–582.

[mpp70135-bib-0053] Levy, C. 2005. “Epidemiology and Chemical Control of Soybean Rust in Southern Africa.” Plant Disease 89: 669–674. 30795397 10.1094/PD-89-0669

[mpp70135-bib-0054] Li, F. , N. M. Upadhyaya , J. Sperschneider , et al. 2019. “Emergence of the Ug99 Lineage of the Wheat Stem Rust Pathogen Through Somatic Hybridisation.” Nature Communications 10: 5068. 10.1038/s41467-019-12927-7 PMC6838127 31699975

[mpp70135-bib-0055] Li, H. , and R. Durbin . 2009. “Fast and Accurate Short Read Alignment With Burrows‐Wheeler Transform.” Bioinformatics 25: 1754–1760. 19451168 10.1093/bioinformatics/btp324 PMC2705234

[mpp70135-bib-0056] Lorrain, C. , K. C. Gonçalves dos Santos , H. Germain , A. Hecker , and S. Duplessis . 2019. “Advances in Understanding Obligate Biotrophy in Rust Fungi.” New Phytologist 222: 1190–1206. 30554421 10.1111/nph.15641

[mpp70135-bib-0057] McClure, M. C. , D. Bickhart , D. Null , et al. 2014. “Bovine Exome Sequence Analysis and Targeted SNP Genotyping of Recessive Fertility Defects BH1, HH2, and HH3 Reveal a Putative Causative Mutation in SMC2 for HH3.” PLoS One 9: e92769. 24667746 10.1371/journal.pone.0092769 PMC3965462

[mpp70135-bib-0058] Minh, B. Q. , H. A. Schmidt , O. Chernomor , et al. 2020. “IQ‐TREE 2: New Models and Efficient Methods for Phylogenetic Inference in the Genomic Era.” Molecular Biology and Evolution 37: 1530–1534. 10.1093/molbev/msaa015. 32011700 PMC7182206

[mpp70135-bib-0059] Mukrimin, M. , A. Kovalchuk , L. G. Neves , et al. 2018. “Genome‐Wide Exon‐Capture Approach Identifies Genetic Variants of Norway Spruce Genes Associated With Susceptibility to *Heterobasidion parviporum* Infection.” Frontiers in Plant Science 9: 793. 29946332 10.3389/fpls.2018.00793 PMC6005875

[mpp70135-bib-0060] Müller, M. A. , G. Stammler , and L. L. May De Mio . 2021. “Multiple Resistance to DMI, QoI and SDHI Fungicides in Field Isolates of *Phakopsora pachyrhizi* .” Crop Protection 145: 105618.

[mpp70135-bib-0061] Murithi, H. M. , J. S. Haudenshield , F. Beed , G. Mahuku , M. Joosten , and G. L. Hartman . 2017. “Virulence Diversity of *Phakopsora pachyrhizi* Isolates From East Africa Compared to a Geographically Diverse Collection.” Plant Disease 101: 1194–1200. 30682948 10.1094/PDIS-10-16-1470-RE

[mpp70135-bib-0062] Murithi, H. M. , R. M. Soares , G. Mahuku , H. P. van Esse , and M. H. Joosten . 2021. “Diversity and Distribution of Pathotypes of the Soybean Rust Fungus *Phakopsora pachyrhizi* in East Africa.” Plant Pathology 70: 655–666.

[mpp70135-bib-0063] Nakamoto, A. A. , P. M. Joubert , and K. V. Krasileva . 2023. “Intraspecific Variation of Transposable Elements Reveals Differences in the Evolutionary History of Fungal Phytopathogen Pathotypes.” Genome Biology and Evolution 15: evad206. 37975814 10.1093/gbe/evad206 PMC10691877

[mpp70135-bib-0105] Nieuwenhuis, B. P. S. , and T. Y. James 2016. “The frequency of sex in fungi.” Philosophical Transactions of the Royal Society B: Biological Sciences 371: 20150540. 10.1098/rstb.2015.0540 PMC5031624 27619703

[mpp70135-bib-0064] Oggenfuss, U. , and D. Croll . 2023. “Recent Transposable Element Bursts Are Associated With the Proximity to Genes in a Fungal Plant Pathogen.” PLoS Pathogens 19: e1011130. 36787337 10.1371/journal.ppat.1011130 PMC9970103

[mpp70135-bib-0065] Oliveira, J. I. N. , and N. Corradi . 2024. “Strain‐Specific Evolution and Host‐Specific Regulation of Transposable Elements in the Model Plant Symbiont *Rhizophagus irregularis* .” G3: Genes, Genomes, Genetics 14: jkae055. 38507600 10.1093/g3journal/jkae055 PMC11075540

[mpp70135-bib-0066] Ono, Y. , P. Buriticá , and J. F. Hennen . 1992. “Delimitation of *Phakopsora*, *Physopella* and *Cerotelium* and Their Species on Leguminosae.” Mycological Research 96: 825–850.

[mpp70135-bib-0067] Paineau, M. , M. Zaccheo , M. Massonnet , and D. Cantu . 2024. “Advances in Grape and Pathogen Genomics Toward Durable Grapevine Disease Resistance.” Journal of Experimental Botany: erae450. 10.1093/jxb/erae450 PMC12321742 39487719

[mpp70135-bib-0068] Pan, Z. , X. B. Yang , S. Pivonia , L. Xue , R. Pasken , and J. Roads . 2006. “Long‐Term Prediction of Soybean Rust Entry Into the Continental United States.” Plant Disease 90: 840–846. 30781018 10.1094/PD-90-0840

[mpp70135-bib-0069] Panstruga, R. 2003. “Establishing Compatibility Between Plants and Obligate Biotrophic Pathogens.” Current Opinion in Plant Biology 6: 320–326. 12873525 10.1016/s1369-5266(03)00043-8

[mpp70135-bib-0070] Pantou, M. P. , V. N. Kouvelis , and M. A. Typas . 2008. “The Complete Mitochondrial Genome of *Fusarium oxysporum*: Insights Into Fungal Mitochondrial Evolution.” Gene 419: 7–15. 18538510 10.1016/j.gene.2008.04.009

[mpp70135-bib-0071] Paul, C. , R. D. Frederick , C. B. Hill , G. L. Hartman , and D. R. Walker . 2015. “Comparison of Pathogenic Variation Among *Phakopsora pachyrhizi* Isolates Collected From the United States and International Locations, and Identification of Soybean Genotypes Resistant to the US Isolates.” Plant Disease 99: 1059–1069. 30695939 10.1094/PDIS-09-14-0989-RE

[mpp70135-bib-0073] Quek, Z. B. R. , and S. H. Ng . 2024. “Hybrid‐Capture Target Enrichment in Human Pathogens: Identification, Evolution, Biosurveillance, and Genomic Epidemiology.” Pathogens 13: 275. 38668230 10.3390/pathogens13040275 PMC11054155

[mpp70135-bib-0074] Radhakrishnan, G. V. , N. M. Cook , V. Bueno‐Sancho , et al. 2019. “MARPLE, a Point‐of‐Care, Strain‐Level Disease Diagnostics and Surveillance Tool for Complex Fungal Pathogens.” BMC Biology 17: 65. 31405370 10.1186/s12915-019-0684-y PMC6691556

[mpp70135-bib-0075] Ristaino, J. B. , P. K. Anderson , D. P. Bebber , et al. 2021. “The Persistent Threat of Emerging Plant Disease Pandemics to Global Food Security.” Proceedings of the National Academy of Sciences of the United States of America 118: e2022239118. 34021073 10.1073/pnas.2022239118 PMC8201941

[mpp70135-bib-0076] Rossi, R. L. 2003. “First Report of *Phakopsora pachyrhizi*, the Causal Organism of Soybean Rust in the Province of Misiones, Argentina.” Plant Disease 87: 102. 10.1094/PDIS.2003.87.1.102A 30812688

[mpp70135-bib-0077] Rush, T. A. , J. Golan , A. McTaggart , C. Kane , R. W. Schneider , and M. C. Aime . 2019. “Variation in the Internal Transcribed Spacer Region of *Phakopsora pachyrhizi* and Implications for Molecular Diagnostic Assays.” Plant Disease 103: 2237–2245. 31306089 10.1094/PDIS-08-18-1426-RE

[mpp70135-bib-0078] Saunders, D. G. O. , Z. A. Pretorius , and M. S. Hovmøller . 2019. “Tackling the Re‐Emergence of Wheat Stem Rust in Western Europe.” Communications Biology 2: 51. 30729187 10.1038/s42003-019-0294-9 PMC6361993

[mpp70135-bib-0079] Savary, S. , L. Willocquet , S. J. Pethybridge , P. Esker , N. McRoberts , and A. Nelson . 2019. “The Global Burden of Pathogens and Pests on Major Food Crops.” Nature Ecology & Evolution 3: 430–439. 30718852 10.1038/s41559-018-0793-y

[mpp70135-bib-0080] Schneider, R. W. , C. A. Hollier , H. K. Whitam , et al. 2005. “First Report of Soybean Rust Caused by *Phakopsora pachyrhizi* in the Continental United States.” Plant Disease 89: 774. 10.1094/PD-89-0774A 30791253

[mpp70135-bib-0081] Schwessinger, B. , Y. J. Chen , R. Tien , et al. 2020. “Distinct Life Histories Impact Dikaryotic Genome Evolution in the Rust Fungus *Puccinia striiformis* Causing Stripe Rust in Wheat.” Genome Biology and Evolution 12, no. 5: 597–617. 32271913 10.1093/gbe/evaa071 PMC7250506

[mpp70135-bib-0082] Sheeja, T. E. , I. P. V. Kumar , A. Giridhari , D. Minoo , M. K. Rajesh , and K. N. Babu . 2021. “Amplified Fragment Length Polymorphism: Applications and Recent Developments.” In Molecular Plant Taxonomy: Methods and Protocols, edited by P. Besse , 187–218. Springer US. 10.1007/978-1-0716-0997-2_12 33301096

[mpp70135-bib-0083] Sikora, E. 2014. “Kudzu: Invasive Weed Supports the Soybean Rust Pathogen Through Winter Months in Southeastern United States.” Outlooks on Pest Management 25: 175–179.

[mpp70135-bib-0085] Sperschneider, J. , T. Hewitt , D. C. Lewis , et al. 2023. “Nuclear Exchange Generates Population Diversity in the Wheat Leaf Rust Pathogen *Puccinia triticina* .” Nature Microbiology 8: 2130–2141. 10.1038/s41564-023-01494-9 PMC10627818 37884814

[mpp70135-bib-0086] Stewart, S. , M. Rodríguez , and N. Yamanaka . 2019. “Pathotypic Variation of *Phakopsora pachyrhizi* Isolates From Uruguay.” Tropical Plant Pathology 44: 309–317.

[mpp70135-bib-0087] Stokstad, E. 2004. “Plant Pathologists Gear Up for Battle With Dread Fungus.” Science 306: 1672–1673. 15576584 10.1126/science.306.5702.1672

[mpp70135-bib-0088] Stone, C. L. , M. L. P. Buitrago , J. L. Boore , and R. D. Frederick . 2010. “Analysis of the Complete Mitochondrial Genome Sequences of the Soybean Rust Pathogens *Phakopsora pachyrhizi* and *P. meibomiae* .” Mycologia 102: 887–897. 20648755 10.3852/09-198

[mpp70135-bib-0089] Sydow, H. , and P. Sydow . 1914. “A Contribution to Knowledge of the Parasitic Fungi on the Island of Formosa.” Annales Mycologici 12: 105.

[mpp70135-bib-0090] Thilliez, G. J. A. , M. R. Armstrong , T.‐Y. Lim , et al. 2019. “Pathogen Enrichment Sequencing (PenSeq) Enables Population Genomic Studies in Oomycetes.” New Phytologist 221: 1634–1648. 30288743 10.1111/nph.15441 PMC6492278

[mpp70135-bib-0091] Torres, D. E. , B. P. H. J. Thomma , and M. F. Seidl . 2021. “Transposable Elements Contribute to Genome Dynamics and Gene Expression Variation in the Fungal Plant Pathogen *Verticillium dahliae* .” Genome Biology and Evolution 13: evab135. 34100895 10.1093/gbe/evab135 PMC8290119

[mpp70135-bib-0092] Twizeyimana, M. , and G. L. Hartman . 2012. “Pathogenic Variation of *Phakopsora pachyrhizi* Isolates on Soybean in the United States From 2006 to 2009.” Plant Disease 96: 75–81. 30731859 10.1094/PDIS-05-11-0379

[mpp70135-bib-0093] Twizeyimana, M. , P. S. Ojiambo , J. S. Haudenshield , et al. 2011. “Genetic Structure and Diversity of *Phakopsora pachyrhizi* Isolates From Soyabean.” Plant Pathology 60: 719–729.

[mpp70135-bib-0094] Twizeyimana, M. , P. S. Ojiambo , K. Sonder , T. Ikotun , G. L. Hartman , and R. Bandyopadhyay . 2009. “Pathogenic Variation of *Phakopsora pachyrhizi* Infecting Soybean in Nigeria.” Phytopathology 99: 353–361. 19271976 10.1094/PHYTO-99-4-0353

[mpp70135-bib-0095] Urquhart, A. S. , N. F. Chong , Y. Yang , and A. Idnurm . 2022. “A Large Transposable Element Mediates Metal Resistance in the Fungus *Paecilomyces variotii* .” Current Biology 32: 937–950.e5. 35063120 10.1016/j.cub.2021.12.048

[mpp70135-bib-0096] van der Auwera, G. , and B. D. O'Connor . 2020. Genomics in the Cloud: Using Docker, GATK, and WDL in Terra. O'Reilly Media, Incorporated.

[mpp70135-bib-0097] Vittal, R. , H.‐C. Yang , and G. L. Hartman . 2012. “Anastomosis of Germ Tubes and Migration of Nuclei in Germ Tube Networks of the Soybean Rust Pathogen, *Phakopsora pachyrhizi* .” European Journal of Plant Pathology 132: 163–167.

[mpp70135-bib-0098] Walker, D. R. , H. R. Boerma , D. V. Phillips , et al. 2011. “Evaluation of USDA Soybean Germplasm Accessions for Resistance to Soybean Rust in the Southern United States.” Crop Science 51: 678–693.

[mpp70135-bib-0099] Wyka, S. , S. Mondo , M. Liu , V. Nalam , and K. Broders . 2022. “A Large Accessory Genome and High Recombination Rates May Influence Global Distribution and Broad Host Range of the Fungal Plant Pathogen *Claviceps purpurea* .” PLoS One 17: e0263496. 35143550 10.1371/journal.pone.0263496 PMC8830672

[mpp70135-bib-0100] Yamaoka, Y. , N. Yamanaka , H. Akamatsu , and K. Suenaga . 2014. “Pathogenic Races of Soybean Rust *Phakopsora pachyrhizi* Collected in Tsukuba and Vicinity in Ibaraki, Japan.” Journal of General Plant Pathology 80: 184–188.

[mpp70135-bib-0101] Yorinori, J. T. , W. M. Paiva , R. D. Frederick , et al. 2005. “Epidemics of Soybean Rust (*Phakopsora pachyrhizi*) in Brazil and Paraguay From 2001 to 2003.” Plant Disease 89: 675–677. 30795398 10.1094/PD-89-0675

[mpp70135-bib-0106] Yu, G. , D. K. Smith , H. Zhu , et al. 2016. “ ggtree: An r Package for Visualization and Annotation of Phylogenetic Trees With Their Covariates and Other Associated Data.” Methods in Ecology and Evolution 8: 28–36.

[mpp70135-bib-0102] Zhang, C. , S. S. Dong , J. Y. Xu , W. M. He , and T. L. Yang . 2019. “PopLDdecay: A Fast and Effective Tool for Linkage Disequilibrium Decay Analysis Based on Variant Call Format Files.” Bioinformatics 35: 1786–1788. 30321304 10.1093/bioinformatics/bty875

[mpp70135-bib-0103] Zhang, X. , M. Freire , M. Le , et al. 2012. “Genetic Diversity and Origins of *Phakopsora pachyrhizi* Isolates in the United States.” Asian Journal of Plant Pathology 6: 52–65.

[mpp70135-bib-0104] Zheng, W. , L. Huang , J. Huang , et al. 2013. “High Genome Heterozygosity and Endemic Genetic Recombination in the Wheat Stripe Rust Fungus.” Nature Communications 4: 2673. 10.1038/ncomms3673 PMC3826619 24150273

