# Supplementary figures and images for: Population Genomics Reveals Distinct Lineage of the Asian Soybean Rust Fungus Phakopsora pachyrhizi in the United States of America Unrelated to Brazilian Populations
Source: Mol Plant Pathol. 2025 Aug 7;26(8):e70135. doi: 10.1111/mpp.70135 (PMC12330935; doi:10.1111/mpp.70135)

(a)

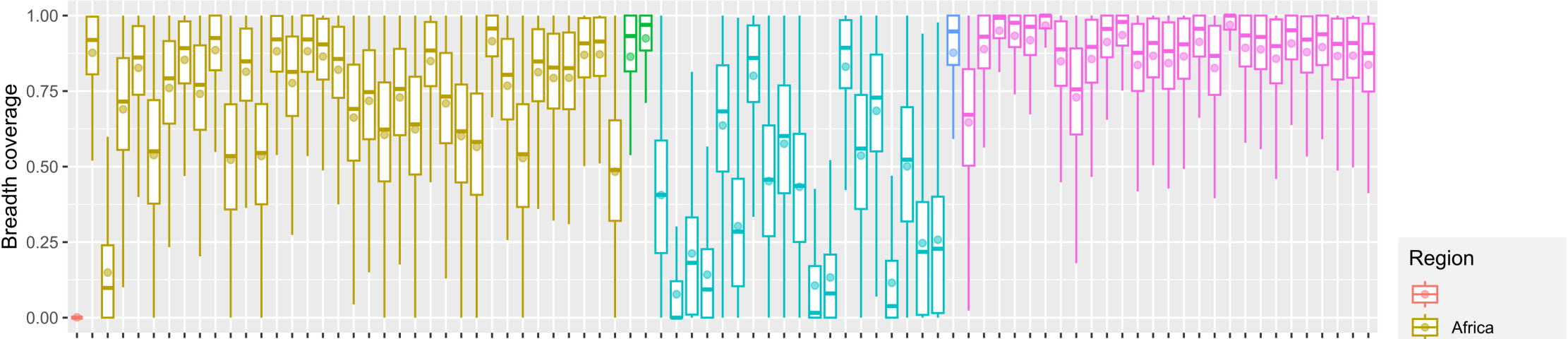

(b)

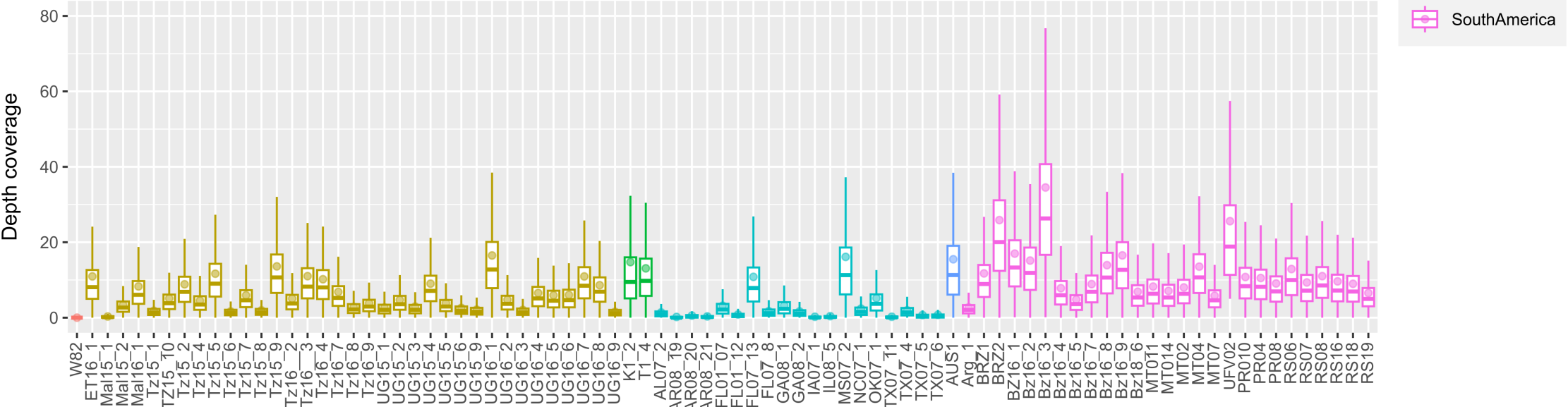

Supplement: Supplementary file 1 — Figure S1: Read breadth and depth coverage of the UFV02 “Gene Catalogue” genes in 85 exome‐capture samples. (a) Read breadth and (b) depth coverage over genic regions of 10,942 protein‐coding genes in the nuclear genome (exome‐captured UFV02 “Gene Catalogue” genes) were calculated and are represented as boxplots. The first sample is W82 (red), a negative control to test the specificity of the exome‐capture array. Outlier points were omitted. Dots indicate mean values. [file MPP-26-e70135-s004.pdf]

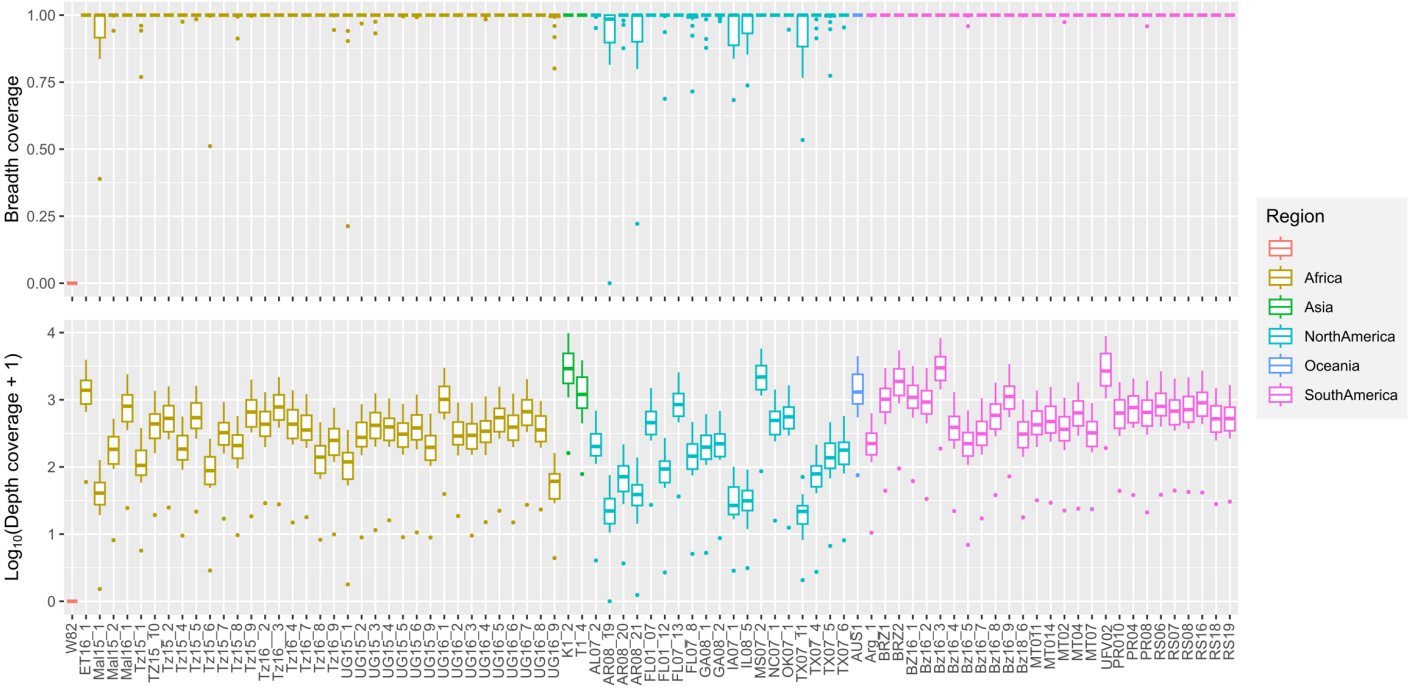

Supplement: Supplementary file 2 — Figure S2: Read breadth coverage of mt genes in 85 exome‐capture samples. (a) Read breadth coverage over genic regions of 15 protein‐coding genes in the mt genome (b) Log10 fold depth coverage of sequencing reads. The first sample is W82 (red), a negative control to test the specificity of the exome‐capture array. Both sequencing read breadth and depth coverage were calculated and are represented as boxplots. [file MPP-26-e70135-s011.pdf]

(a)

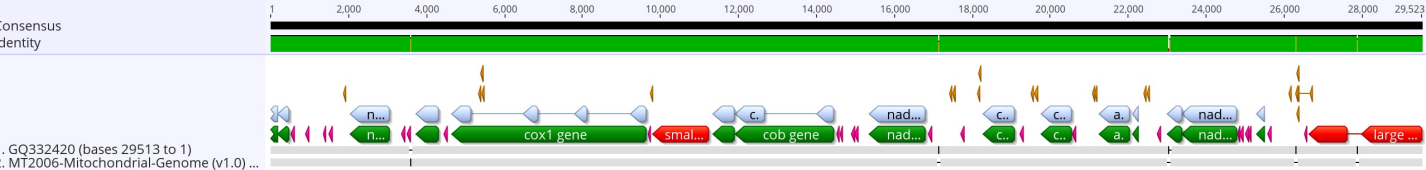

(b)

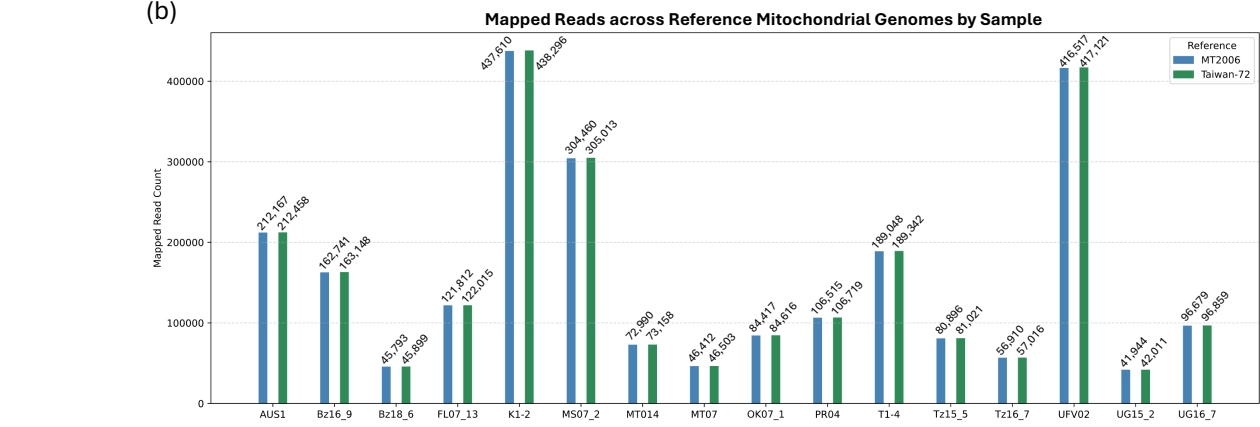

(c)

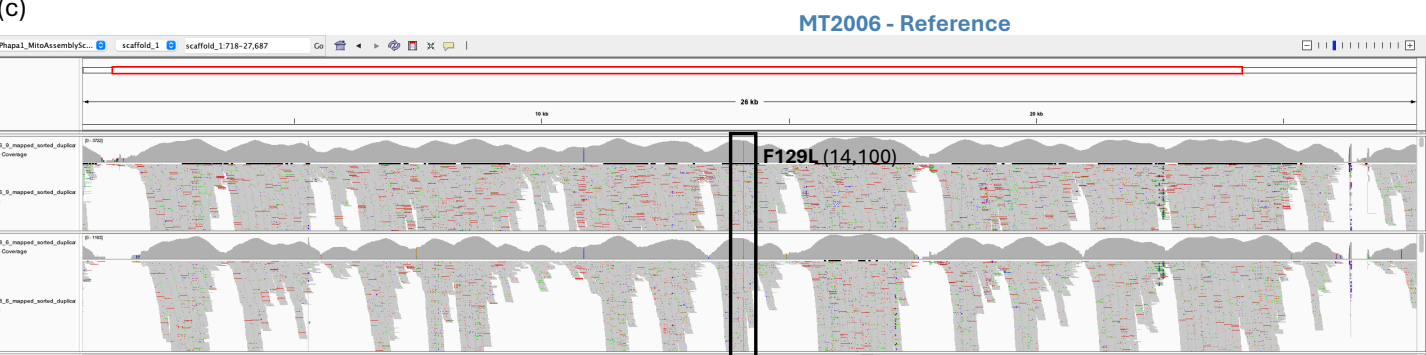

(d)

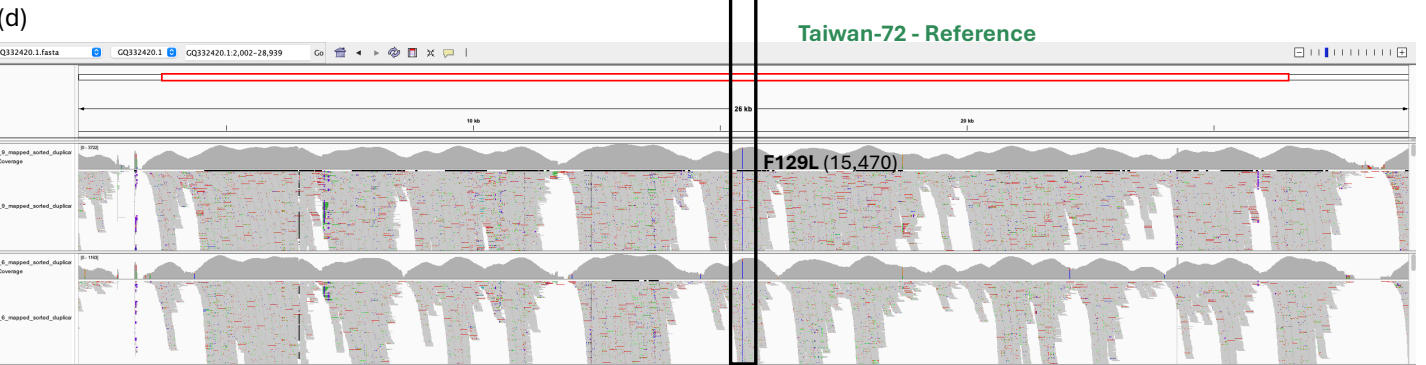

Supplement: Supplementary file 3 — Figure S3: Genomic comparison between mitochondrial genomes of the isolates Taiwan‐72 and MT2006. (a) Whole genome alignment performed by Geneious software (MAUVE alignment) showing highly conservation between the Taiwan‐72 genome (Genbank CG332420) and the MT2006 genome. (b) Number of mapped reads across different mt reference genomes in samples from South America (Bz_16_9; Bz_18_6; MT014; MT07; PR04, UFV02); Africa (Tz15_5; Tz16_7; UG15_2; UG16_7); United States of America (Fl07_13; MS07_2; OK07_1); Australia (AUS1); and Japan (K1‐2; T1‐4). Screenshots of IGV software showing that the mutation F129L present in Brazilian isolates is identified using both the (c) Taiwan‐72 and (d) MT2006 reference genomes. [file MPP-26-e70135-s008.pdf]

Mean depth coverage

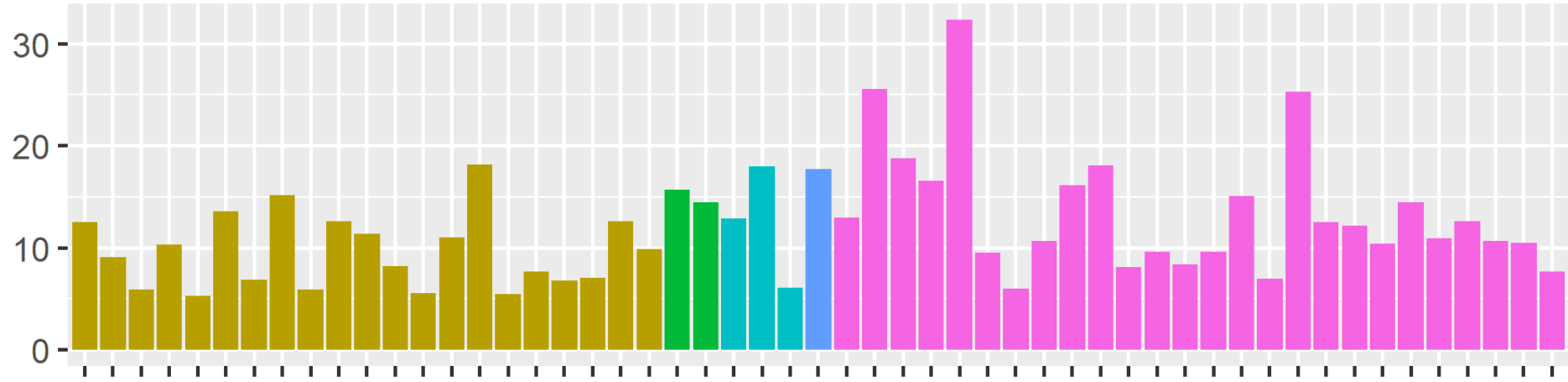

Region

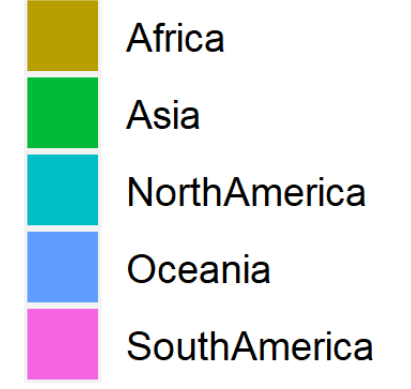

Count

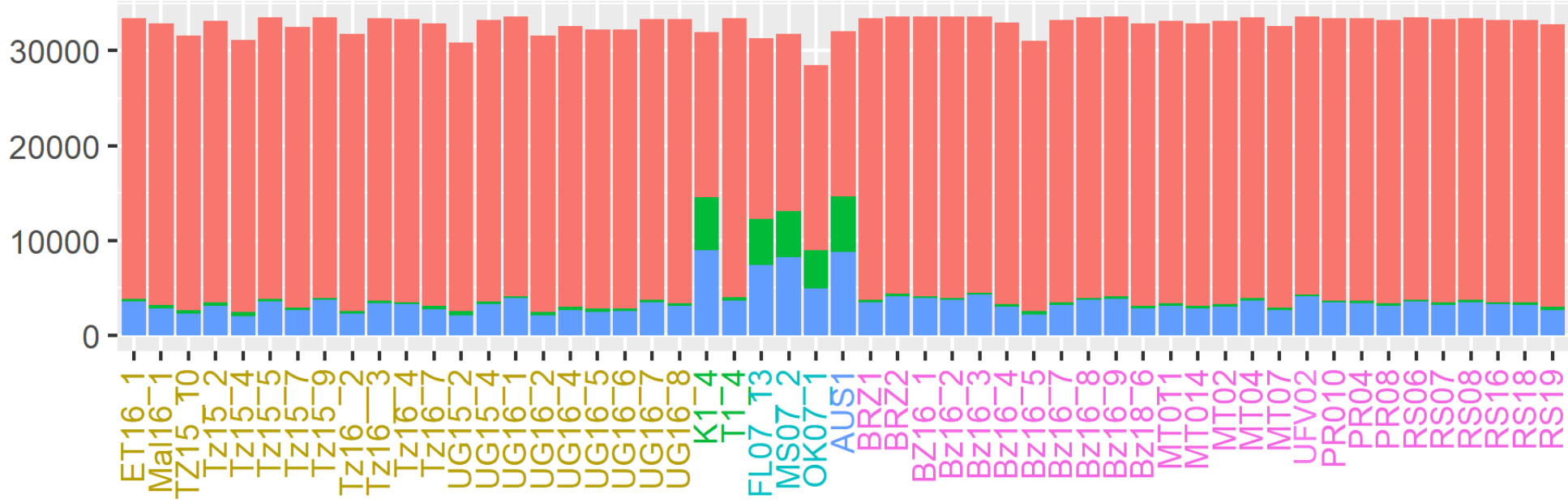

Genotype

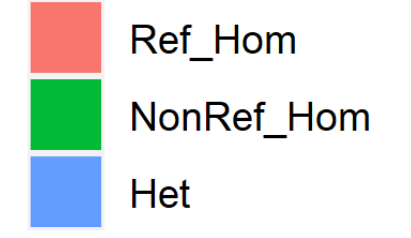

Sample\_id

Supplement: Supplementary file 4 — Figure S4: Read breadth coverage and genotypes of SNP sites in the nuclear genome in 53 exome‐capture samples. [file MPP-26-e70135-s006.pdf]

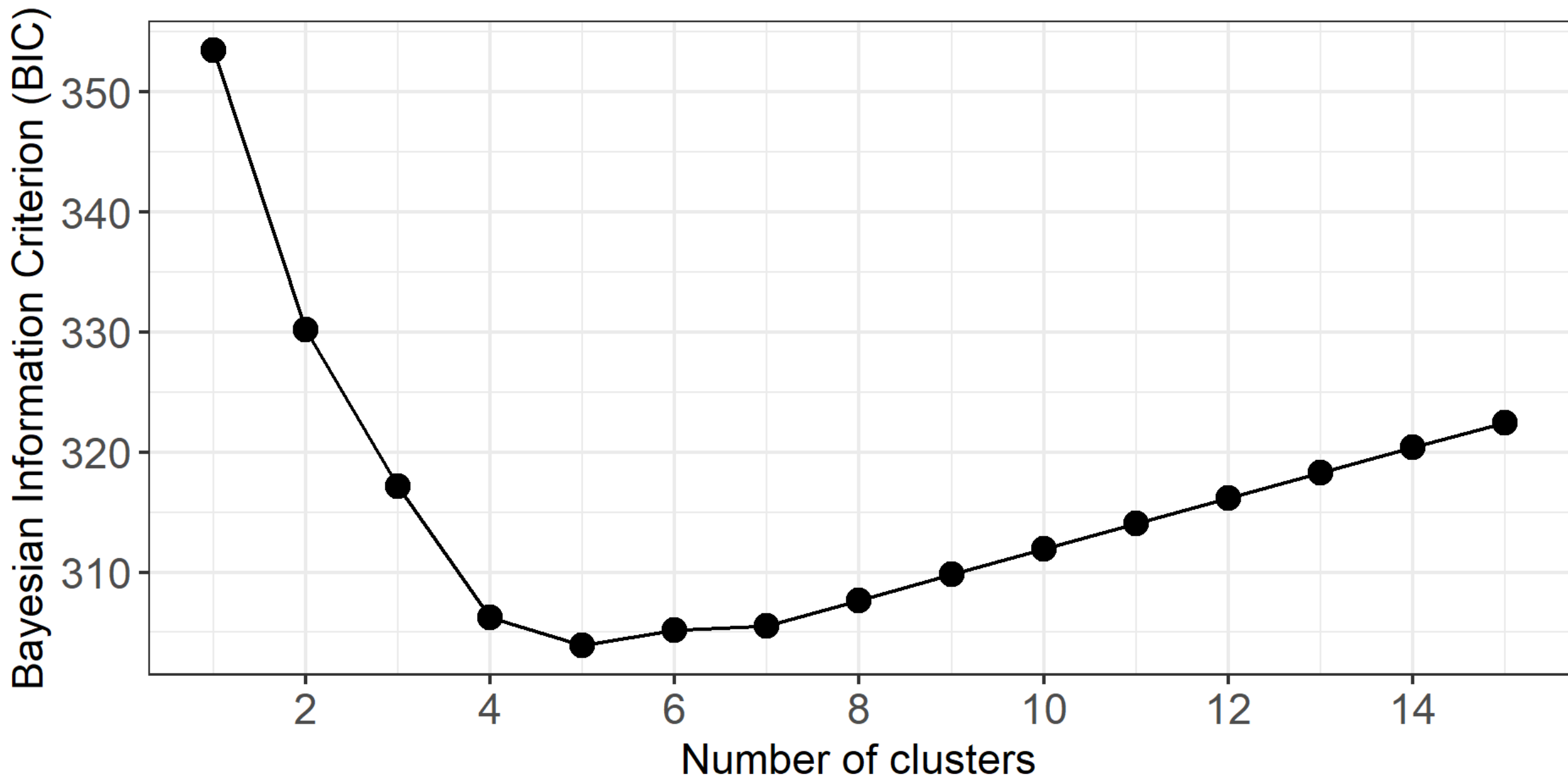

Supplement: Supplementary file 5 — Figure S5: Elbow plot of Bayesian Information Criterion (BIC) for different number of clusters, k. To identify the optimal number of clusters, k‐means was run sequentially with increasing values of k, and different clustering solutions were compared using BIC. [file MPP-26-e70135-s007.pdf]

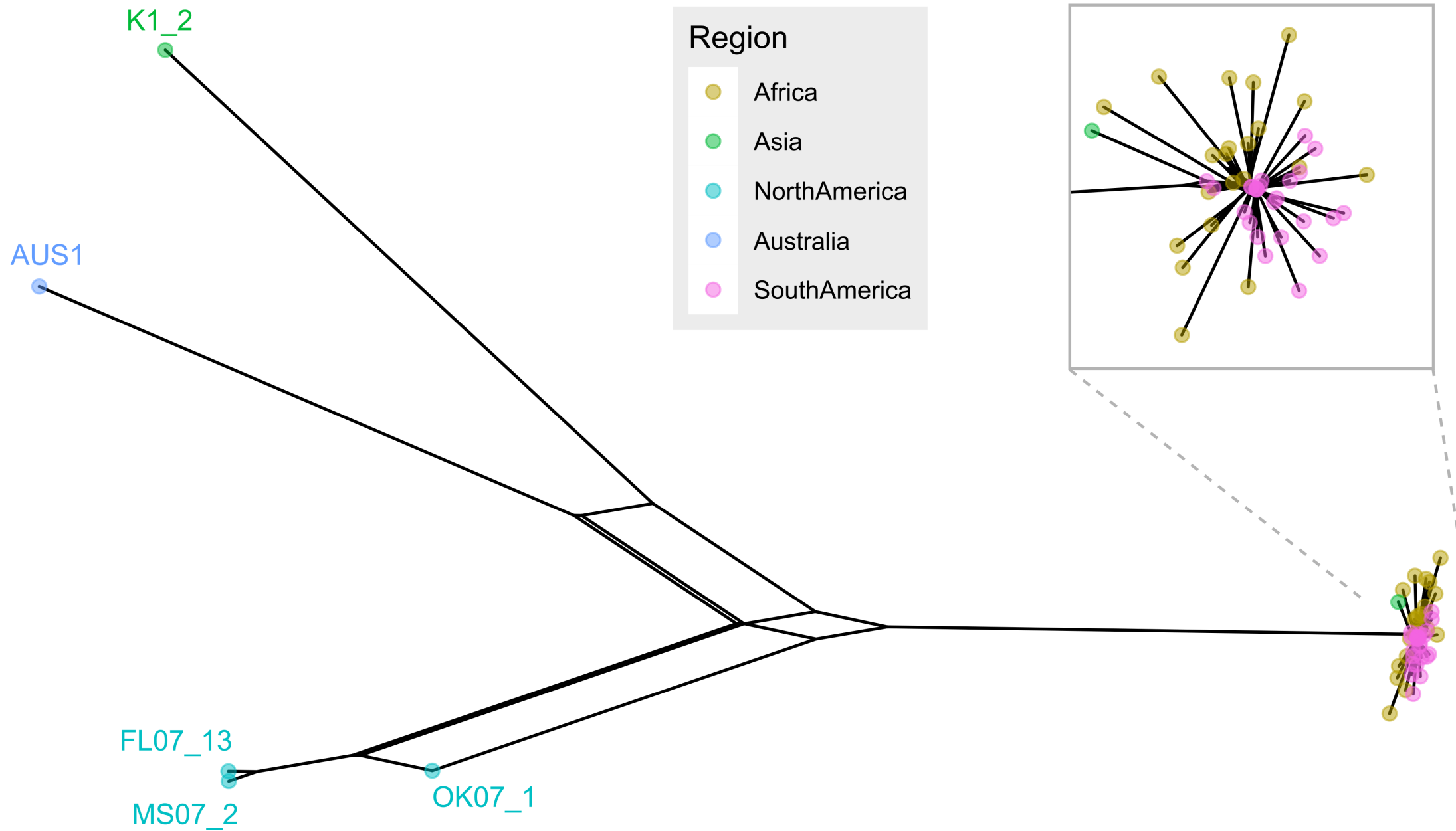

Supplement: Supplementary file 6 — Figure S6: A neighbour‐net phylogenetic network of P. pachyrhizi samples inferred from nuclear genome SNPs. A phylogenetic network was inferred from 33,634 SNP markers with hamming distance and neighborNet function implemented in the phangorn package in R. Dots represent the samples and are colour‐coded on the basis of the geographic origin of the samples. [file MPP-26-e70135-s001.pdf]

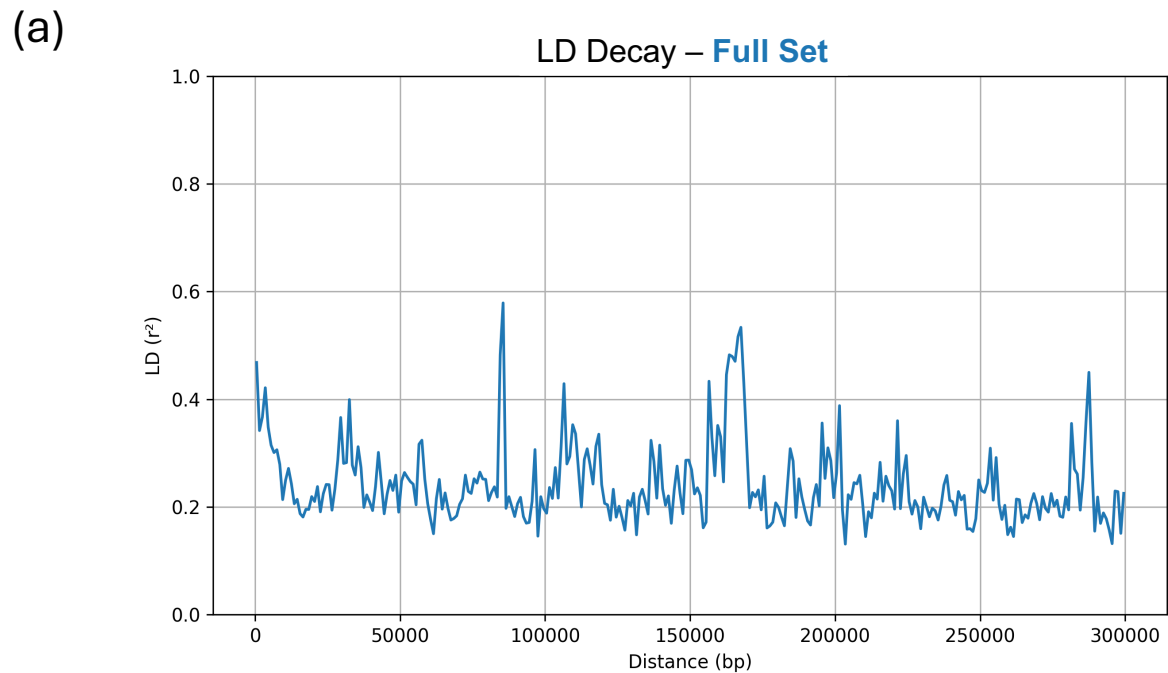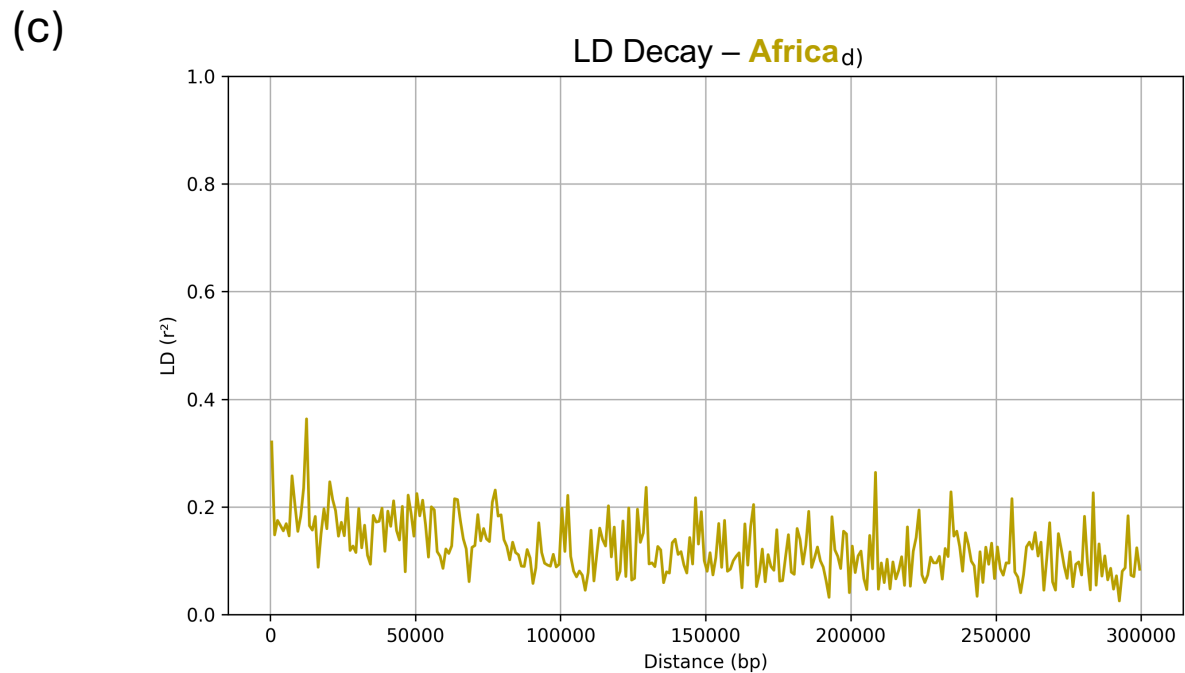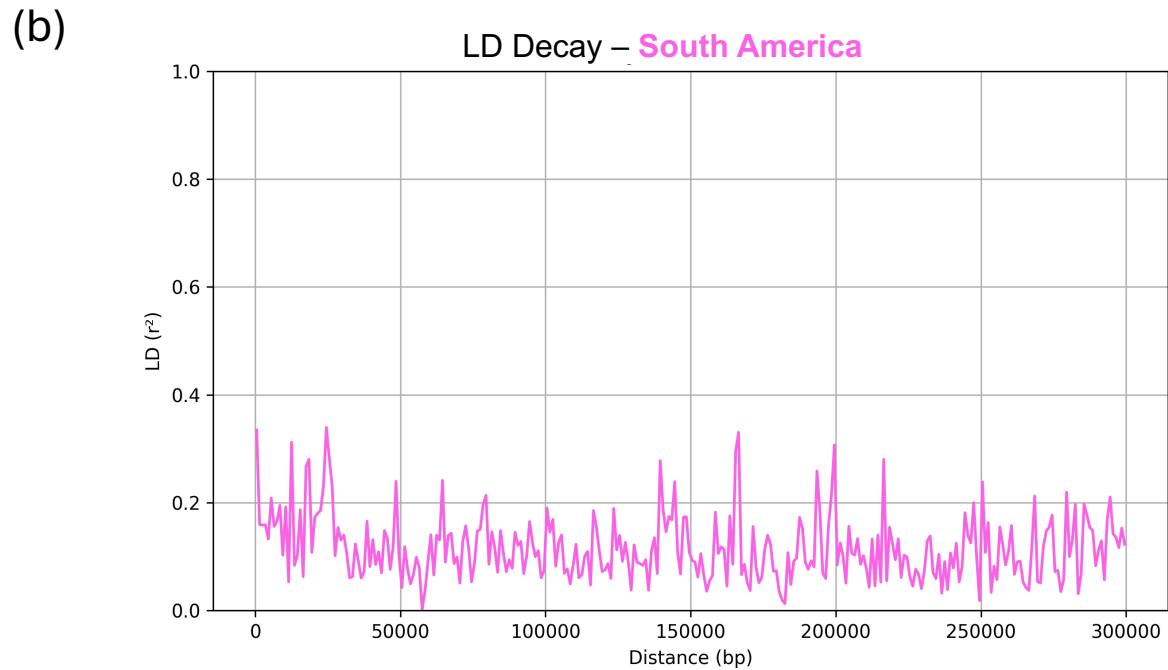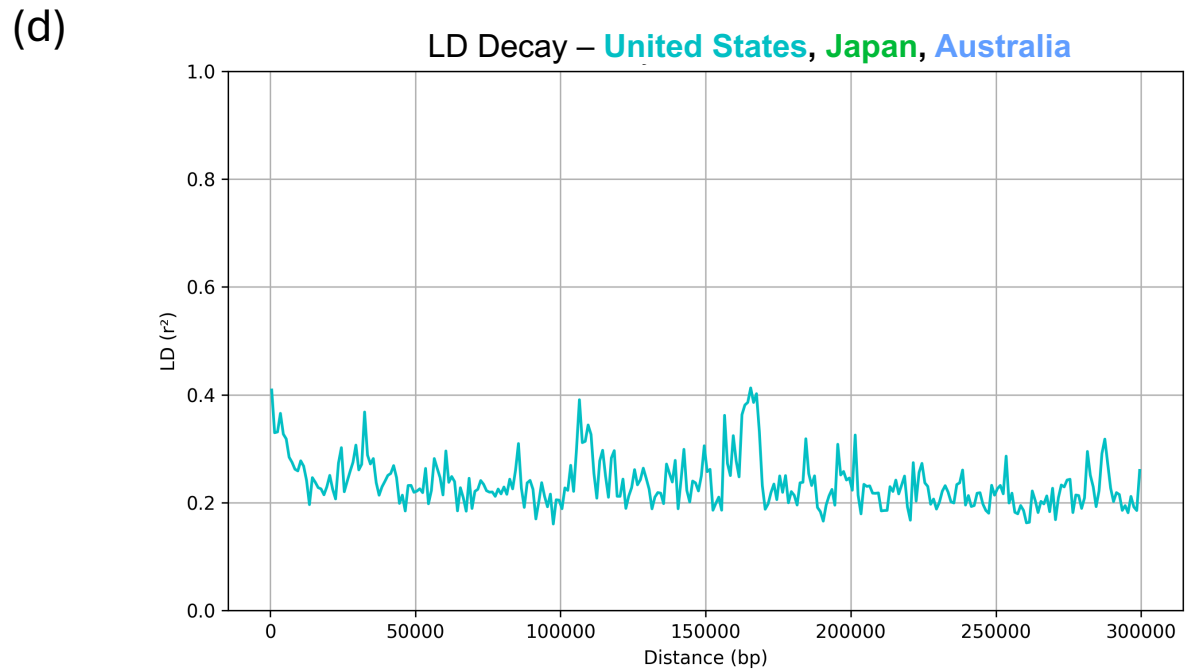

Supplement: Supplementary file 7 — Figure S7: Linkage disequilibrium (LD) patterns in the full set of 53 isolates of P. pachyrhizi. Squared correlation coefficient (r 2) values are represented in the y‐axis and physical distance between pairs of SNPs are represented in the x‐axis. (a) LD decay patterns of the full set of 53 isolates. (b) South America sub‐set calculated with 26 isolates. (c) LD decay patterns of Africa sub‐set (21 isolates). (d) LD decay of United States, Japan and Australia isolates (5 isolates). [file MPP-26-e70135-s005.pdf]
